# Evaluation method for asymmetric uncertainty of quantitative polymerase chain reaction measurements of deoxyribonucleic acids with low copy number

**DOI:** 10.1038/s41598-021-90959-0

**Published:** 2021-06-02

**Authors:** Unoh Ki, Takeru Suzuki, Satoshi Nakazawa, Yuuki Yonekawa, Kazuki Watanabe, Michie Hashimoto, Shigeo Hatada, Hirotaka Unno

**Affiliations:** Biomedical Business Center, Healthcare Business Group, Ricoh Company, Ltd., 3-25-22 Tonomachi LIC 322, Kawasaki, Kanagawa 210-0821 Japan

**Keywords:** DNA, Target validation, Target identification, Genetic engineering

## Abstract

Recently, in food safety and various other fields, qualitative and quantitative gene analysis using real-time polymerase chain reaction (PCR) method has become increasingly popular. The limit of detection (LOD) and quantifiable range for these measurements depends on the range and precision of DNA calibrators’ concentrations. Low-copy-number nucleic acid reference materials with low uncertainty produced by an inkjet system have been developed to allow for precise measurements in a low-copy-number region. However, when using a calibrator with a low copy number near one, the copy number distribution is asymmetric. Consequently, the confidence intervals of estimated copy numbers can include negative values when conventional methods of uncertainty estimation are used. A negative confidence interval is irrelevant in the context of copy number, which is always positive value or zero. Here, we propose a method to evaluate the uncertainty of real-time PCR measurements with representative values and an asymmetric 95% confidence interval. Moreover, we use the proposed method for the actual calculation of uncertainty of real-time PCR measurement results for low-copy-number DNA samples and demonstrate that the proposed method can evaluate the precision of real-time PCR measurements more appropriately in a low-copy-number region.

## Introduction

The PCR method developed by Mullis in 1983^1^, a technique to amplify small amounts of DNA for analysis, has been broadly applied in both science and engineering as well as in agriculture and medicine. In particular, the spread of the quantitative PCR method has greatly contributed to the development of various fields that require the quantitative analysis of trace amounts of nucleic acids, including the field of food inspection^2–6^, such as genetically modified foods and microbiological analysis; the field of laboratory tests^7^, such as cancer and infection; and the field of forensic medicine^8^.

Because PCR has become increasingly popular worldwide, the appropriate quality control of PCR-based analytical methods has become necessary, and international standardization activities have been particularly active in recent years. ISO20395 states the importance of evaluating the limit of detection (LOD) for qualitative analysis of the target nucleic acid sequence or the limit of quantification (LOQ) for quantitative analysis as well as understanding the performance of the PCR system, including reagents and instruments, before analysis^9^. This can be interpreted to indicate that the performance of the reaction system should be assessed using calibrators (reference nucleic acid materials) covering a full range of concentrations to be quantified to obtain more reliable analysis results from quantitative real-time PCR^10,11^ analysis with extremely small amounts of nucleic acids^12^. To meet such demand, we developed a new cell-based DNA reference material (hereinafter referred to as the new reference material) containing the target DNA sequence as low as one copy^13^. Note that “copy” is a unit generally used to express one DNA molecule containing a certain sequence.

The uncertainty of real-time PCR measurements is primarily composed of two components, calibrator uncertainty and measurement process uncertainty; both these components are assumed to follow a normal distribution^14,15^. Therefore, measurement results are represented by the mean DNA concentration of each sample and symmetrical measurement uncertainty. However, in an extremely low concentration range, quantitative analysis has a problem that a confidence limit of DNA concentration can be a negative value. Therefore, methods to impart more probable uncertainty information are required. One solution is to measure the same sample enough number of times to reduce measurement uncertainty. However, in reality, it is desirable to have a method to evaluate the uncertainty information efficiently and reproducibly with at most 4–5 repeat measurements. This study reports that the inclusion of asymmetry in uncertainty can provide more reliable uncertainty information in an extremely low concentration range with one copy.

## Results

In this study, we compared the new method of estimating asymmetric uncertainty with the conventional method of uncertainty estimation based on normal distribution. After real-time PCR quantification experiments, the uncertainty of quantification results was estimated with the two methods. To demonstrate the versatility of the new method, we created calibration curves with two different calibrators: one prepared by the conventional dilution method and another prepared with the new reference material.

### Sources of the uncertainty of real-time PCR

As mentioned above, the uncertainty of real-time PCR measurements has the following two major components: (1) uncertainty of calibrator copy number and (2) uncertainty from the measurement process. Generally, the calibrator uncertainty is combined with the uncertainty of the original solution’s concentration used to prepare serial dilutions and the uncertainty of the measuring instrument used for dilution. After one step of dilution, the measuring instrument’s uncertainty is from a total of two measurements, i.e., one from the original solution and another from the buffer used for dilution. It is desirable to select an appropriate range and avoid using an excessively small volume for measurement to reduce the uncertainty of the measuring instrument^12,14^. Furthermore, when low-copy-number samples are used, the variability in original solution sampling becomes significant because of an effect of the Poisson distribution^16^ and is desirable to consider as the third source of uncertainty^12,17^. However, the new reference material was prepared using inkjet to dispense a cell suspension into a container to yield a specified copy number. We considered the specified number of copies to be dispensed as a measured value and calculated the uncertainty of measurements from the production variability and the number of samples used. In addition to the variability of the copy number of intracellular target DNA and the variability because of contamination, which have been evaluated in the previous study^13^, cell aggregation was identified to be another source of the production variability. To increase the production efficiency, two avalanche photodiodes (APD410A/M, Thorlabs; hereinafter referred to as APD) placed oppositely were used to detect any cells in flying droplets (Figure S1 in Supplementary Information File 1). APDs convert detected fluorescent energies of cells into voltage values. When output voltage value exceeded the threshold (set to 0.2 V), it was considered that a cell was detected. Count errors occur depending on the detection rate (probability of detecting droplets containing a particle) and false detection rate (probability of detecting particles that are not cells) of APD as well as the possibility that one droplet contains two or more cells (dependent on the cell suspension concentration). Therefore, the variation because of APD count errors was calculated. The term DNA below always refers to DNA molecules containing the target sequence.

### The conventional method to calculate the uncertainty of concentrations in a serial dilution

We calculated the symmetric uncertainty of the concentrations of serially diluted, certified DNA reference material as described in the Guide to the Expression of Uncertainty in Measurement^18^ (Sect. Determining combined standard uncertainty), briefly introduced in Supplementary Information File 2. Table 1 shows all variables and parameters used in this paper.

**Table 1:** List of variables and parameters.

Variable	Definition	Equation no.
*C*_*after*_ (unit: μL^−1^)	DNA concentration of diluted solution after each dilution step	(1)
*C*_*before*_ (unit: μL^−1^)	DNA concentration of the original solution at each dilution step	(1)
*P* (unit: μL)	Volume of the original solution at each dilution step	(1)
*Q* (unit: μL)	Volume of the buffer added at each dilution step	(1)
*E*	Error of DNA copy number in the *P* volume of the original solution due to the Poisson distribution at each dilution step	(1)
*N*_*P*_	DNA copy number in the *P* volume of the original solution at each dilution step	
*e*	Specific value of *E*	(2)
*c*_*after*_ (unit: μL^−1^)	Estimate of the expectation of *C*_*after*_	(2)
*c*_*before*_ (unit: μL^−1^)	Estimate of the expectation of *C*_*before*_	(2)
*p* (unit: μL)	Estimate of the expectation of *P*	(2)
*q* (unit: μL)	Estimate of the expectation of *Q*	(2)
*u*(*c*_*after*_)	Symmetric uncertainty of *c*_*after*_	(2)
*u*(*c*_*before*_)	Symmetric uncertainty of *c*_*before*_	(2)
*u*(*p*)	Symmetric uncertainty of *p*	(2)
*u*(*q*)	Symmetric uncertainty of *q*	(2)
*u*(*e*)	Symmetric uncertainty of *e*	(2)
*c*_*1*_	Sensitivity coefficient of *C*_*before*_	(2), (5) and (6)
*c*_*2*_	Sensitivity coefficient of *P*	(2), (5) and (6)
*c*_*3*_	Sensitivity coefficient of *Q*	(2), (5) and (6)
*c*_*4*_	Sensitivity coefficient of *E*	(2), (5) and (6)
*N*_*dil*_	DNA copy number of a calibrator prepared with dilution series in a well	
$\overline{{N }_{dil}}$	Mean of *N*_*dil*_ in each well	(3)
$\overline{{n }_{dil}}$	Estimate of the expectation of $\overline{{N }_{dil}}$	
*C*_*final*_ (unit: μL^−1^)	DNA concentration of a diluted solution used to prepare a calibrator	(3)
*P*_*final*_ (unit: μL)	Volume of a diluted solution dispensed in each well to prepare a calibrator	(3)
*E*_*final*_	Error of DNA copy number of a calibrator prepared with dilution series due to the Poisson distribution	
*e*_*final*_	Specific value of *E*_*final*_	
$\overline{{E }_{final}}$	Mean of *E*_*final*_ in each well	(3)
*c*_*final*_ (unit: μL^−1^)	Estimate of the expectation of *C*_*final*_	(4)
*p*_*final*_ (unit: μL)	Estimate of the expectation of *P*_*final*_	(4)
$\overline{{e }_{final}}$	Estimate of the expectation of $\overline{{E }_{final}}$	(4)
*u*($\overline{{n }_{dil}}$)	Symmetric uncertainty of $\overline{{n }_{dil}}$	(4)
*u*(*c*_*final*_)	Symmetric uncertainty of *c*_*final*_	(4)
*u*(*p*_*final*_)	Symmetric uncertainty of *p*_*final*_	(4)
*u*($\overline{{e }_{final}}$)	Symmetric uncertainty of $\overline{{e }_{final}}$	(4)
*c*_*5*_	Sensitivity coefficient of *C*_*final*_	(4)
*c*_*6*_	Sensitivity coefficient of *P*_*final*_	(4)
*c*_*7*_	Sensitivity coefficient of $\overline{{E }_{final}}$	(4)
*N*_*rw*_	Effective number of wells containing the same specific calibrator	
*A*_*c_after*_, *B*_*c_after*_	Left- and right-side confidence intervals for the expectation of *C*_*after*_	(5) and (6)
*A*_*c_before*_, *B*_*c_before*_	Left- and right-side confidence intervals for the expectation of *C*_*before*_	(5) and (6)
*A*_*p*_, *B*_*p*_	Left- and right-side confidence intervals for the expectation of *P*	(5) and (6)
*A*_*q*_, *B*_*q*_	Left- and right-side confidence intervals for the expectation of *Q*	(5) and (6)
*A*_*e*_, *B*_*e*_	Left and right intervals for representing the asymmetric variety of *E*	(5) and (6)
${A}_{\overline{n\_dil}}$, ${B}_{\overline{n\_dil}}$	Left- and right-side confidence intervals for the expectation of $\overline{{n }_{dil}}$	(7) and (8)
*A*_*c_final*_, *B*_*c_final*_	Left- and right-side confidence intervals for the expectation of *C*_*final*_	(7) and (8)
*A*_*p_final*_, *B*_*p_final*_	Left- and right-side confidence intervals for the expectation of *P*_*final*_	(7) and (8)
${A}_{\overline{e\_final}}$, ${B}_{\overline{e\_final}}$	Left- and right-side confidence intervals for the expectation of $\overline{{E }_{final}}$	(7) and (8)
*P*_*agg*_	Cell aggregation rate	
*N*_*agg*_	Number of masses of cell aggregate containing ≥ 2 cells when measuring *P*_*agg*_	
*N*_*sgl*_	Number of independent cell when measuring *P*_*agg*_	
*P*_*D*_	Detection rate of the APDs corresponding to detect a fluorescent particle (either a cell of a fluorescent contaminant) in a droplet	(9) and (11)
*P*_*FD*_	False detection rate of the APDs corresponding to detect a fluorescent contaminant in a droplet	(10)
*N*_1_	Number of droplets containing one cell aggregate (both a independent cell and a cell aggregate containing 2 cells are referred to as a cell aggregate) when measuring *P*_*D*_ and *P*_*FD*_	(9) and (10)
*N*_*APD_*0_	Number of times the APDs detected droplets containing 0 cell aggregates	(9) and (10)
*N*_*APD_*1_	Number of times the APDs detected droplets containing 1 cell aggregate	(9) and (10)
*λ*_*pa*_	Mean fluorescent particle number in each droplet	(11)
*P*_*pa_*0_	Proportion of droplets containing no fluorescent particle	(11)
*N*_*pos*_	Number of droplets in which APD detected particles when measuring *λ*_*pa*_	(11)
*N*_*all*_	Total number of droplets when measuring *λ*_*pa*_	(11)
*P*_*con*_	Probability of well contamination by DNA	
*P*_*intra_*2_	Proportion of single cells with an intracellular DNA copy number of two	
${\widehat{P}}_{pa\_0}$	Worst value of *P*_*pa_*0_	
*N*_*pa_droplet*_	Number of fluorescent particles in a droplet	(12)
*N*_*pa*_	Number of particles dispensed between two consecutive APD detections	(12)
*n*_*pa*_	Specific value of *N*_*pa*_	(12)
${\widehat{P}}_{D}$	Worst value of *P*_*D*_	(12)
${\widehat{\lambda }}_{pa}$	Worst value of *λ*_*pa*_	
*N*_*new*_	DNA copy number of a calibrator prepared with the new reference material in each well	
$\overline{{N }_{new}}$	Mean of *N*_*new*_ in each well	(13)
*N*_*APD*_	Predetermined number of APD detections in each well set in the inkjet system	(13)
$\overline{{N }_{pa}}$	Mean of *N*_*pa*_	(13)
$\overline{{N }_{agg\_pa}}$	Mean number of cell aggregates in a fluorescent particle	(13)
$\overline{{N }_{sgl\_agg}}$	Mean number of cells in a cell aggregate	(13)
$\overline{{N }_{intra}}$	Mean intracellular DNA copy number	(13)
$\overline{{N }_{con}}$	Mean contaminant DNA copy number in a well from reagents and environment	(13)
$\overline{{n }_{new}}$	Estimate of the expectation of $\overline{{N }_{new}}$	(14)
$u\left(\overline{{n }_{new}}\right)$	Symmetric uncertainty of $\overline{{n }_{new}}$	(14)
${\sigma }_{N\_new}$	Standard deviation of *N*_*new*_	(14)
*A*_*n_new*_, *B*_*n_new*_	Left and right intervals for representing the asymmetric variety of *N*_*new*_	(15) and (16)
${A}_{\overline{n\_new}}$, ${B}_{\overline{n\_new}}$	Left and right confidence intervals for the expectation of $\overline{{N }_{new}}$	(15) and (16)
*u*(*e*_*curve*_)	Symmetric uncertainty of a calibration curve in real-time PCR process	(17)
*e*_*curve*_	Estimation of the error of the mean quantification result sourced from the calibration curve	(17)
$\overline{{n }_{Q}}$	Mean PCR quantification result of replicates of target samples	(17)
*N*_*rw,i*_	Effective number of wells containing calibrators with the same *i*-th representative DNA copy number	(17)
$\overline{{n }_{r,i}}$	Estimate of mean DNA copy number of calibrators with the same *i*-th representative DNA copy number	(17)
${R}_{\overline{n\_r,i}}$	Representative value of $\overline{{n }_{r,i}}$	(17)
$u\left(\overline{{n }_{r,i}}\right)$	Symmetric uncertainty of $\overline{{n }_{r,i}}$	(17)
*s*_*n_rQ,i*_	Corrected sample standard deviation of PCR quantification results of replicates of calibrators with the same *i*-th representative DNA copy number	(17)
$\overline{{n }_{rQ,i}}$	Mean of PCR quantification results of replicates of calibrators with the same *i*-th representative DNA copy number	(17)
*U*_*conv*_	Symmetric expanded uncertainty of the mean DNA copy number of target samples in each well	(18)
*s*_*n_Q*_	Corrected sample standard deviation of real-time PCR quantification results of replicates of target samples	(18)
*N*_*w*_	Number of wells containing replicates of target samples including those failed in amplification (undetermined)	(18)
${t}_{0.05,{N}_{w}-1}$	Two-tailed t critical value	(18)
*X*	Lognormal distributed variable, corresponding to the PCR quantification result of either a calibrator or a target sample	
$\overline{X}$	Mean of *X*	(19) and (20)
*s*_*X*_	Corrected sample standard deviation of *X*	(19) and (20)
*Y*	Natural logarithm of *X*	
*s*_*Y*_	Corrected sample standard deviation of *Y*	(19)
$\overline{Y}$	Mean of *Y*	(20)
${A}_{\overline{X} }$, ${B}_{\overline{X} }$	Left- and right-side confidence intervals for $\overline{X}$	(21) and (22)
*M*_*X*_	Median of *X*	(21–23)
*A*_*e_curve*_, *B*_*e_curve*_	Left- and right-side confidence intervals for the expectation of *e*_*curve*_	(24) and (25)
*M*_*Q*_	Estimated median of PCR quantification results of replicates of target samples	(24) and (25)
${A}_{\overline{n\_r,i}}$, ${B}_{\overline{n\_r,i}}$	Left- and right-side confidence intervals for the expectation of $\overline{{n }_{r,i}}$	(24) and (25)
*M*_*rQ,i*_	Estimated median of PCR quantification results of replicates of calibrators with the same *i*-th representative DNA copy number	(24) and (25)
${A}_{\overline{n\_rQ,i}}$, ${B}_{\overline{n\_rQ,i}}$	Left- and right-side confidence intervals for the expectation of $\overline{{n }_{rQ,i}}$	(24) and (25)
*A*_*prop*_, *B*_*prop*_	Left and right confidence intervals for the expectation of the mean DNA copy number of target samples	(26) and (27)
*A*_*Q*_, *B*_*Q*_	Left and right confidence intervals of the mean PCR quantification result of replicates of target samples	(26) and (27)

The concentration *C*_*after*_ (unit: μL^−1^) after each dilution step is described using the following equation:

1 $$\begin{array}{c}{C}_{after}=\frac{P{\cdot C}_{before}+E}{P+Q}\end{array}$$

In this equation, *C*_*before*_ is the DNA concentration of the original solution at each step (unit: μL^−1^); *P* and *Q* are volumes of the original solution and the buffer (unit: μL), respectively; and *E* is the error of DNA copy number. The probability mass function of *E* was defined by *P*_*E*_(*e*) = Pr(*E* = *e*) = Pr(*N*_*P*_ = *e* + *λ*), where *e* is a specific value of *E*, and *N*_*P*_ denotes the DNA copy number in the *P* volume of original solution. *N*_*P*_ is generally assumed to follow the Poisson distribution with an expectation *λ* = *p* · *c*_*before*_, where *p* and *c*_*before*_ are estimates of the expectations of *P* and *C*_*before*_, respectively. Therefore, the expectation of *E* is zero. Note that because dilution was performed serially, *C*_*before*_ at each step is equivalent to *C*_*after*_ at the previous step. Equation (1) corresponds to *f* in equation (S1) in Supplementary Information File 2.

According to the combined uncertainty formula using the sensitivity coefficient of the Guide to the Expression of Uncertainty in Measurement^18^, based on equation (S1), the following equation was used to calculate the uncertainty *u*(*c*_*after*_) of a concentration after dilution:

2 $$\begin{array}{c}u({c}_{after})=\sqrt{{\left[{c}_{1}\cdot u\left({c}_{before}\right)\right]}^{2}+{\left[{c}_{2}\cdot u\left(p\right)\right]}^{2}+{\left[{c}_{3}\cdot u\left(q\right)\right]}^{2}+{\left[{c}_{4}\cdot u\left(e\right)\right]}^{2}}\\ =\sqrt{{\left[\frac{p}{p+q}\cdot u\left({c}_{after}\right)\right]}^{2}+{\left[\frac{q}{{\left(p+q\right)}^{2}}{c}_{before}\cdot u\left(p\right)\right]}^{2}+{\left[-\frac{p}{{\left(p+q\right)}^{2}}{c}_{before}\cdot u\left(q\right)\right]}^{2}+{\left[\frac{1}{p+q}\cdot u\left(e\right)\right]}^{2}}\end{array}$$

In this equation, *c*_*after*_ and *q* are estimates of expectations of *C*_*after*_ and *Q*; *u*(*c*_*before*_), *u*(*p*), *u*(*q*), and *u*(*e*) are the uncertainties of their respective variables; and *c*_*1*_, *c*_*2*_, *c*_*3*_, and *c*_*4*_ are sensitivity coefficients of *C*_*before*_, *P*, *Q*, and *E*, respectively. Because *p* and *q* can only be measured once, the only component of *u*(*p*) and *u*(*q*) is the uncertainty of the pipette. Therefore *u*(*p*) and *u*(*q*) are obtained directly from the pipette calibration certificate. At each dilution step, *u*(*c*_*before*_) equals *u*(*c*_*after*_) calculated in the previous step. The volume of the original solution was dispensed once at each step. Therefore, *u*(*e*) was the standard deviation of the Poisson distribution ${\sigma }_{Poisson}=\sqrt{p{\cdot C}_{before}}$. Note that *u*(*c*_*before*_) at the first step is the uncertainty of the certified reference material, which is 9.75 × 10^7^ μL^−1^ according to the manufacturer's certificate.

We used these calculations to determine the concentration uncertainties of a series of diluted DNA solutions.

### A conventional method to calculate the uncertainty of DNA copy number prepared by dispensing a dilution series

We calculated the symmetric uncertainty of the mean DNA copy number in calibrators prepared by dispensing a diluted solution of a specific concentration into each well. After preparing the dilution series, 4 μL of each diluted solution was dispensed into a 96-well plate with a pipette. Therefore, the mean final copy number *N*_*dil*_ in each well, $\overline{{N }_{dil}}$, is described using the following equation, corresponding to *f* in equation (S1):

3 $$\begin{array}{c}\overline{{N }_{dil}}={P}_{final}\cdot {C}_{final}+\overline{{E }_{final}}\end{array}$$

In this equation, *C*_*final*_ (unit: μL^−1^) is the concentration of the diluted solution in a container to be dispensed into each well; *P*_*final*_ (unit: μL) is the volume of solution dispensed in each well, set to 4 μL; and $\overline{{E }_{final}}$ is the mean error of DNA copy number *E*_*final*_ in each well. The probability mass function of *E*_*final*_ was defined by *P*_*E_final*_(*e*_*final*_) = Pr(*E*_*final*_ = *e*_*final*_) = Pr(*N*_*dil*_ = *e*_*final*_ + *λ*_*final*_), where *e*_*final*_ is a specific value of *E*_*final*_, and *N*_*dil*_ follows the Poisson distribution with an expectation *λ*_*final*_ = *p*_*final*_ ∙ *c*_*final*_. Here, *p*_*final*_ and *c*_*final*_ are estimates of the expectations of *P*_*final*_ and *C*_*final*_, respectively. Moreover, *p*_*final*_ was much smaller than the remaining volume of the diluted solution in the container. Thus, *c*_*final*_ was assumed to remain the same throughout dispensing. Furthermore, dispensing was performed independently for each well. Therefore, *N*_*dil*_ in each well was assumed to independently and identically follow the Poisson distribution with the same *λ*_*final*_. Note that *c*_*final*_ corresponds to *c*_*after*_ calculated at a certain dilution step.

Based on equation (S1), the uncertainty of the mean of the final copy number in each well *u*($\overline{{n }_{dil}}$) was calculated using the following equation:

4 $$\begin{array}{c}u\left(\overline{{n }_{dil}}\right)=\sqrt{{\left[{c}_{5}\cdot u\left({c}_{final}\right)\right]}^{2}+{\left[{c}_{6}\cdot u\left({p}_{final}\right)\right]}^{2}+{\left[{c}_{7}\cdot u\left(\overline{{e }_{final}}\right)\right]}^{2}}\\ =\sqrt{{\left[{p}_{final}\cdot u\left({c}_{final}\right)\right]}^{2}+{\left[{c}_{final}\cdot u\left({p}_{final}\right)\right]}^{2}+{u}^{2}\left(\overline{{e }_{final}}\right)}\end{array}$$

In this equation, $\overline{{n }_{dil}}$ and $\overline{{e }_{final}}$ are estimates of expectations of $\overline{{N }_{dil}}$ and $\overline{{E }_{final}}$, respectively; *u*(*c*_*final*_), *u*(*p*_*final*_), and *u*($\overline{{e }_{final}}$) are uncertainties of their respective variables, respectively; and *c*_*5*_, *c*_*6*_, and *c*_*7*_ are sensitivity coefficients of *C*_*final*_, *P*_*final*_, and $\overline{{E }_{final}}$, respectively. The only component of *u*(*p*_*final*_) was the uncertainty of the pipette, which can be obtained directly from the calibration certificate. Moreover, the standard error of the mean $\overline{{e }_{final}}$, ${\sigma }_{{\overline{e\_final}}}=\sqrt{({p}_{final}\cdot {c}_{final})/{N}_{rw}}$, corresponds to *u*($\overline{{e }_{final}}$). Here, *N*_*rw*_ is the effective number of wells containing the same specific calibrator. The number of wells that failed to amplify is excluded. An alternative model for calculating *u*($\overline{{n }_{dil}}$) is introduced in Supplementary Information File 2 and assumes that *c*_*final*_ and *p*_*final*_ were correlated in all wells.

We used these calculations to determine the uncertainties of the mean DNA copy number of calibrators prepared by dispensing 4 μL of diluted solution at different concentrations into each well, $u\left(\overline{{n }_{dil}}\right)$. A representative value is defined in this paper to represent the measurement results of a variable, generally expressed as a mean. The representative value of the mean copy number $\overline{{n }_{dil}}$ was *p*_*final*_ ∙ *c*_*final*_.

### A proposed method to calculate the uncertainty of a serial dilution

We calculated asymmetric uncertainties of concentration in serial dilutions and the mean DNA copy number of calibrators prepared by dispensing diluted solution into independent wells, compared to the symmetric uncertainty calculated above.

First, we used an asymmetric 95% confidence interval for each component to estimate the uncertainty of *C*_*after*_. We assumed an asymmetric 95% confidence interval could be denoted by [*R* − *A*, *R* + *B*]. *R* denotes the representative value. *A* and *B* denote the left-side confidence interval (the interval between the representative value and the lower confidence limit) and the right-side confidence interval (the interval between the representative value and the upper confidence limit). Therefore, *A* and *B* represent the asymmetric expanded uncertainty of the variable. Because it was not considered proper to calculate expanded uncertainty using a cover factor when a variable is not normally distributed, we estimated the expanded uncertainty of each variable separately.

The left- and right-side confidence intervals for the expectation of *C*_*after*_, *A*_*c_after*_ and *B*_*c_after*_, were calculated separately based on Eq. (2), using the following equations:

5 $$\begin{array}{c}{A}_{c\_after}=\sqrt{{\left({c}_{1}\cdot {A}_{c\_before}\right)}^{2}+{\left({c}_{2}\cdot {A}_{p}\right)}^{2}+{\left({c}_{3}\cdot {A}_{q}\right)}^{2}+{\left({c}_{4}\cdot {A}_{e}\right)}^{2}}\end{array}$$

6 $$\begin{array}{c}{B}_{c\_after}=\sqrt{{\left({c}_{1}\cdot {B}_{c\_before}\right)}^{2}+{\left({c}_{2}\cdot {B}_{p}\right)}^{2}+{\left({c}_{3}\cdot {B}_{q}\right)}^{2}+{\left({c}_{4}\cdot {B}_{e}\right)}^{2}}\end{array}$$

In these equations, *A*_*c_before*_ and *B*_*c_before*_ denote left- and right-side confidence intervals for the expectation of *C*_*before*_; *A*_*p*_ and *B*_*p*_ denote left- and right-side confidence intervals for the expectation of *P*; *A*_*q*_ and *B*_*q*_ denote left- and right-side confidence intervals for the expectation of *Q*; and *A*_*e*_ and *B*_*e*_ denote left- and right-side intervals representing the asymmetric variety of *E*. At each dilution step, *A*_*c_before*_ and *B*_*c_before*_ are *A*_*c_after*_ and *B*_*c_after*_ of the previous step. The concentration of the certified reference material was assumed to follow a normal distribution. Therefore, *A*_*c_before*_ and *B*_*c_before*_ at the first dilution step were approximated to be twice the uncertainty obtained from the manufacturer’s certificate. In addition, the volume of the solution measured and dispensed by a pipette is also generally assumed to follow a normal distribution. Thus, *A*_*p*_ and *B*_*p*_, and *A*_*q*_ and *B*_*q*_ were approximated with 2*u*(*p*) and 2*u*(*q*), respectively. Furthermore, *A*_*e*_ and *B*_*e*_ were asymmetrically evaluated as shown in Figure S2 in Supplementary Information File 1. The probability mass function of *E* was *P*_*E*_(*e*) defined above. The representative value of *e* was the expectation 0, and the initial value of *A*_*e*_ and *B*_*e*_ defined in Figure S2 were both 0. We then compared the probability that *e* would fall within the interval [− *A*_*e*_, *B*_*e*_] when *A*_*e*_ was increased by a step value of 1 (because *e* is an integer) with that when *B*_*e*_ was increased by 1 and updated the interval to the one with a higher probability. This process was repeated until the probability reached or exceeded 95%. Note that the representative value of *c*_*after*_ was the estimate of the expectation, which was equal to $\frac{p{\cdot c}_{before}}{p+q}$.

As the asymmetric expanded uncertainty of each serially diluted solution was calculated, we then calculated the asymmetric expanded uncertainty for the expectation of the mean DNA copy number of calibrators in each well prepared by dispensing a specific diluted solution. The left- and right-side confidence intervals for the expectation of $\overline{{N }_{dil}}$, ${A}_{\overline{n\_dil}}$ and ${B}_{\overline{n\_dil}}$, were calculated separately based on Eq. (4) using the following equations:

7 $$\begin{array}{c}{A}_{\overline{n\_dil}}=\sqrt{{\left({p}_{final}\cdot {A}_{c\_final}\right)}^{2}+{\left({c}_{final}\cdot {A}_{p\_final}\right)}^{2}+{A}_{\overline{e\_final}}^{2}}\end{array}$$

8 $$\begin{array}{c}{B}_{\overline{n\_dil}}=\sqrt{{\left({p}_{final}\cdot {A}_{c\_final}\right)}^{2}+{\left({c}_{final}\cdot {B}_{p\_final}\right)}^{2}+{B}_{\overline{e\_final}}^{2}}\end{array}$$

In these equations, *A*_*c_final*_ and *B*_*c_final*_ denote left- and right-side confidence intervals for the expectation of *C*_*final*_; *A*_*p_final*_ and *B*_*p_final*_ denote left- and right-side confidence intervals for the expectation of *P*_*final*_; and ${A}_{\overline{e\_final}}$ and ${B}_{\overline{e\_final}}$ denote left- and right-side confidence intervals for the expectation of $\overline{{E }_{final}}$. *A*_*c_final*_ and *B*_*c_final*_ correspond to *A*_*c_after*_ and *B*_*c_after*_ of the specific diluted solution. Moreover, *p*_*final*_ is generally assumed to follow a normal distribution. Therefore, *A*_*p_final*_ and *B*_*p_final*_ were approximated with 2*u*(*p*_*final*_) and ${A}_{\overline{e\_final}}$ and ${B}_{\overline{e\_final}}$ were approximated with ${A}_{e}/\sqrt{{N}_{rw}}$ and ${B}_{e}/\sqrt{{N}_{rw}}$. The approximation of ${A}_{\overline{e\_final}}$ and ${B}_{\overline{e\_final}}$ was similar to the calculation for the standard error of the mean; however, the right and left sides were calculated separately. Note that the representative value $\overline{{n }_{dil}}$ is the mean DNA copy number in each well equal to *p*_*final*_*∙C*_*final*_*.*

We thus obtained asymmetric expanded uncertainties of mean DNA copy number of calibrators prepared by dispensing 4 μL of different concentrations into each well separately. The asymmetric expanded uncertainties were evaluated as ${A}_{\overline{n\_dil}}$ and ${B}_{\overline{n\_dil}}$, compared to symmetric uncertainties evaluated as ${u}_{\overline{n\_dil}}$. Note that symmetric uncertainties were converted to expanded uncertainties in the later calculations of uncertainties in the PCR process.

### Measurement of the production variability of the new reference material

Compared with a calibrator prepared by serial dilution, we calculated the uncertainty when the new reference material was used as a calibrator. To obtain the new reference material’s production variability and uncertainty more precisely, we measured distributions of different influential factors of the production variability. We then used these distributions to estimate the DNA copy number distribution of the calibrator. First, we will describe the measurement of distributions of factors influencing production variability.

The cell aggregation rate in the cell suspension to be dispensed by inkjet was measured using Countess Cell Counting Chamber Slides (Thermo Fisher Scientific) and a microscope (Axio Observer.D1, Carl Zeiss). A 10-μL-sized cell suspension was dispensed into one of the chambers of a slide, and cells in the chamber were imaged using a microscope. Then, the number of masses of aggregated cells, *N*_*agg*_, and the number of independent cells, *N*_*sgl*_, in all images were counted by visual inspection. The aggregation rate *P*_*agg*_ was defined as the ratio of *N*_*agg*_ to the sum of *N*_*agg*_ and *N*_*sgl*_.

The intracellular DNA copy number distribution was measured in the same manner as in the previous study^13^, although a flow cytometer (MA900, Sony) was used for the measurement. In flow cytometry, a cell containing two nuclei cannot be distinguished from an aggregate of two cells containing one nucleus per cell. Therefore, the aggregation rate was subtracted from the proportion of cells with two copies of DNA measured using flow cytometry. This aggregation rate had a different value from the above-mentioned aggregation rate because different processes were used to prepare cell suspensions.

The APD’s detection rate and false detection rate were obtained as follows. A cell suspension was ejected onto an SD00011 slide glass (Matsunami Glass Ind.) by inkjet, and flying droplets were measured using two APDs. Then, the droplets that landed were observed using a microscope to count cell aggregates in each droplet (for convenience of explanation, hereinafter an independent cell is also referred to as an aggregate). To acquire a sufficient number of data, the concentration of particles in a droplet was adjusted to 0.566 (equivalent to 1.48 × 10^6^ mL^−1^). For actual production, the concentration was lower (≤ 0.1); thus, the probability of 2 or more cell aggregates detected in a droplet was very low (≤ 5%). The total fluorescence intensity increased when the droplet contains two or more cell aggregates, and thus the detection rate of APD increases. Therefore, only droplets containing ≤ 1 cell were evaluated in this experiment. The detection rate *P*_*D*_ and the false detection rate *P*_*FD*_ were defined using the following equations:

9 $$\begin{array}{c}{P}_{D}=\frac{{{N}_{APD\_1}+N}_{APD\_0}}{{N}_{1}+{N}_{APD\_0}}\end{array}$$

10 $$\begin{array}{c}{P}_{FD}=\frac{{N}_{APD\_0}}{{N}_{1}+{N}_{APD\_0}}\end{array}$$

In these equations, *N*_1_ was the number of droplets containing one cell aggregate, and *N*_*APD_*0_ and *N*_*APD_*1_ are the number of times the APDs detected droplets containing 0 and 1 cell aggregate, respectively.

False detection events occur primarily because of contamination by fluorescent substances during cell suspension preparation. Therefore, 1 − *P*_*FD*_ represents the proportion of cell aggregates among all fluorescent particles in the cell suspension.

The mean particle number in each droplet was calculated based on the APD measurements of droplets ejected by the inkjet system. The number of particles in each droplet *N*_*pa_droplet*_ (both cell aggregate or fluorescent contaminant count) is generally assumed to follow the Poisson distribution, so the expectation *λ*_*pa*_ is equivalent to the mean particle number. Thus, the proportion of droplets containing no particle *P*_*pa_*0_ was an estimate of the Poisson distribution probability when *N*_*pa_droplet*_ = 0. The probability is equal to exp(− *λ*_*pa*_) based on the definition of the Poisson distribution^19^. Therefore, *λ*_*pa*_ was calculated using the following equation:

11 $$\begin{array}{c}{\lambda }_{pa}=-{\mathrm{ln}}{P}_{pa\_0}=-{\mathrm{ln}}\left(1-\frac{{N}_{pos}}{{N}_{all}}\cdot \frac{1}{{P}_{D}}\right)\end{array}$$

In this equation, *N*_*pos*_ is the number of droplets in which APD detected particles, and *N*_*all*_ is the total number of droplets. The detection rate of APD was also considered in the calculation of *P*_*pa_*0_.

Moreover, we measured contamination by DNA containing the target sequence from reagents and the environment. First, 16 wells of an empty 96-well plate were covered with a seal as negative controls. The plate was then left in the inkjet device without dispensing droplets for the same length of time as normal production. After the seal was removed, cell wall lysis solution was added to all wells as in normal production, and the amplification reagent was added to measure the DNA copy number using real-time PCR. The probability of well contamination by DNA, *P*_*con*_, was then calculated as the ratio of the number of positive wells out of 80 wells that were not covered using a seal.

Moreover, to consider each component’s measurement uncertainty in the calculation, the mean level for each component was set to the worst value in which the uncertainty was considered as follows. First, the measurement of the three components, the intracellular DNA copy number, and the detection and false detection rates of APD, are Bernoulli trials, which follow the Bernoulli distribution^20,21^. Experientially, the aggregates of cells used were mostly composed of two cells, and the contaminant DNA copy number was ≤ 1 in most cases; therefore, the Bernoulli trial was used to approximate these components. Measuring the mean particle number in each droplet follows the Bernoulli distribution and depends on the binary question of whether or not a droplet contains any particles. The parameters of the six Bernoulli distributions described above were *P*_*intra_*2_ denoting the proportion of single cells with an intracellular DNA copy number of 2, the detection rate *P*_*D*_, the false detection rate *P*_*FD*_, the aggregation rate *P*_*agg*_, the probability of DNA contamination *P*_*con*_, and the proportion of droplets with no particles *P*_*pa_*0_. The confidence interval of each of the six parameters was estimated using the Wilson score interval with continuity correction^22^. Finally, the worst values of *P*_*intra_*2_, *P*_*FD*_, *P*_*agg*_, and *P*_*con*_ were set to the corresponding upper confidence limits. The worst values of *P*_*D*_ and *P*_*pa_*0_ were set to the corresponding lower confidence limits. Therefore, the worst value of *λ*_*pa*_ was calculated as $-{\mathrm{ln}}{\widehat{P}}_{pa\_0}$ based on Eq. (11), where ${\widehat{P}}_{pa\_0}$ denote the worst value of *P*_*pa_*0_.

Next, we calculated the distribution of the number of particles dispensed between two consecutive APD detections. After each APD detection, several droplets are usually dispensed before another droplet containing fluorescent particles are detected. Because the detection rate is not 100%, there is a small probability that some particles are present in the undetected droplets. The following equation gives the probability mass function of the total number of particles *N*_*pa*_ in the detected droplet and undetected droplets *P*_*N_pa*_(*n*_*pa*_):

12 $$\begin{array}{c}{P}_{N\_pa}\left({n}_{pa}\right)=Pr\left({N}_{pa}={n}_{pa}\right)=\sum_{i=1}^{{n}_{pa}}\left[{\left(1-{\widehat{P}}_{D}\right)}^{i-1}\cdot {\widehat{P}}_{D}\cdot \frac{{\mathrm{Pr}}{\left({N}_{pa\_droplet}={n}_{pa}-\left(i-1\right)\right)}}{{1-{\mathrm{Pr}}\left({N}_{pa\_droplet}=0\right)}}\right]\end{array}$$

In this equation, *n*_*pa*_ is a specific value of *N*_*pa*_, and ${\widehat{P}}_{D}$ is the worst value of *P*_*D*_ calculated above. *N*_*pa_droplet*_ was assumed to follow the Poisson distribution with an expectation ${\widehat{\lambda }}_{pa}$, where ${\widehat{\lambda }}_{pa}$ is the worst value of *λ*_*pa*_ calculated by using Eq. (11). Experientially, the droplets not detected by APD never contained ≥ 2 particles. Therefore, the number of particles in these droplets was assumed to be 1. Thus, if *n*_*pa*_ − (*i* − 1) particles were contained in the detected droplet, then (*i* − 1) droplets with a single particle were undetected. The item $\frac{{\mathrm{Pr}}{\left({N}_{pa\_droplet}={n}_{pa}-\left(i-1\right)\right)}}{{1-{\mathrm{Pr}}\left({N}_{pa\_droplet}=0\right)}}$ corresponds to the probability that *n*_*pa*_ − (*i* − 1) particles exist in the detected droplet. Note that the number of fluorescent contaminants was included in *n*_*pa*_. The influence of fluorescent contaminants is considered next.

Using the above measurement and calculations, we obtained the distributions of the number of particles dispensed between two consecutive APD detections *N*_*pa*_, the number of cell aggregates in a fluorescent particle determined by *P*_*FD*_, the number of cells in an aggregate determined by *P*_*agg*_, the copy number of intracellular DNA in a cell, and the number of DNA contaminants from reagents and the environment determined by *P*_*con*_.

### Uncertainty of the new reference material

We combined distributions of factors influencing production variability to calculate the uncertainty of the new reference material.

First, we calculated the probability mass function of the DNA copy number of the calibrator prepared with the new reference material, *N*_*new*_. The mean value of *N*_*new*_ in each well, $\overline{{N }_{new}}$, is given by the following equation:

13 $$\begin{array}{c}\overline{{N }_{new}}={N}_{APD}\cdot \left[\left(\overline{{N }_{pa}}\cdot \overline{{N }_{agg\_pa}}\right)\cdot \left(\overline{{N }_{sgl\_agg}}\cdot \overline{{N }_{intra}}\right)\right]+\overline{{N }_{con}}\end{array}$$

In this equation, the constant *N*_*APD*_ is the predetermined number of APD detections in each well set in the inkjet system, $\overline{{N }_{pa}}$ is the mean of *N*_*pa*_, $\overline{{N }_{agg\_pa}}$ is mean number of cell aggregates in a particle, $\overline{{N }_{sgl\_agg}}$ is the mean number of cells in a cell aggregate, $\overline{{N }_{intra}}$ is the mean intracellular DNA copy number, and $\overline{{N }_{con}}$ is the mean contaminant DNA copy number in a well from reagents and environment. Based on the relationship between *N*_*new*_ and factors influencing production variability indicated in Eq. (13), we combined the distributions of these factors and obtained the probability mass function of *N*_*new*_. The method for this calculation is detailed in Supplementary Information File 2.

Next, we calculated the symmetric uncertainty of the mean DNA copy number of the calibrator in each well. The symmetric uncertainty $u\left(\overline{{n }_{new}}\right)$ is the standard error of the mean given by the following equation:

14 $$\begin{array}{c}u\left(\overline{{n }_{new}}\right)={\sigma }_{N\_new}/\sqrt{{N}_{rw}}\end{array}$$

In this equation, $\overline{{n }_{new}}$ is an estimate of the expectation of $\overline{{N }_{new}}$, ${\sigma }_{N\_new}$ is the standard deviation of *N*_*new*_, from equation (S12) in Supplementary Information File 2, and *N*_*rw*_ is the effective number of wells containing the same calibrator.

Finally, as a comparison, we calculated asymmetric expanded uncertainties of the mean DNA copy number of the calibrator in each well. First, left and right intervals representing the asymmetric variety of *N*_*new*_, *A*_*n_new*_ and *B*_*n_new*_, were asymmetrically calculated (Figure S2). The representative value used in the calculation was the median of *N*_*new*_. We considered the median to be a more appropriate representation of an asymmetrically distributed variable, because the median is closer to the peak of the distribution. Note that this interval was an integer because *N*_*new*_ is an integer in principle. Therefore, the interval was symmetric even when the distribution of *N*_*new*_ was asymmetric. Finally, the asymmetric expanded uncertainties for $\overline{{N }_{new}}$, represented by the left and right confidence intervals ${A}_{\overline{n\_new}}$ and ${B}_{\overline{n\_new}}$, were calculated separately (Eq. 14), using the following equations:

15 $$\begin{array}{c}{A}_{\overline{n\_new}}={A}_{n\_new}/\sqrt{{N}_{rw}}\end{array}$$

16 $$\begin{array}{c}{B}_{\overline{n\_new}}={B}_{n\_new}/\sqrt{{N}_{rw}}\end{array}$$

We thus obtained the symmetric uncertainty $u\left(\overline{{n }_{new}}\right)$, and asymmetric expanded uncertainties ${A}_{\overline{N\_new}}$ and ${B}_{\overline{N\_new}}$ of the mean DNA copy number of the calibrator prepared with new reference material in each well. These uncertainties were compared with those of calibrators prepared by serial dilution. Note that the median of *N*_*new*_ was selected to be the representative value of $\overline{{N }_{new}}$.

### Uncertainty of real-time PCR measurement results

Here we describe the calculation of symmetric and asymmetric expanded uncertainties of the mean DNA copy number of target samples in each well quantified by real-time PCR. Relative quantification was performed by plotting calibration curves using calibrators with different representative DNA copy numbers, prepared by serial dilution or new reference material. Quantification with a calibration curve is described in Supplementary Information File 2.

The combined uncertainty was calculated from the mean quantification result and pooled relative uncertainties of several components in a previous study^14^. Here we used the same approach to combine the relative uncertainties of several components. However, the uncertainty of the mean quantification result of DNA copy number of target samples to be quantified was considered to have two major components. The first is the uncertainty of the calibration curve, and the second is the variance between target sample replicates. The calibration curve uncertainty was proposed to have two components: the uncertainty of calibrators prepared by serial dilution or new reference material and the variance in the results of calibrators with the same representative DNA copy number quantified by PCR. Note that DNA copy number of replicates of calibrators were quantified by using the calibration curve plotted by themselves to evaluate the PCR amplification variability of calibrators.

The symmetric expanded uncertainty of the mean DNA copy number of target samples in each well was calculated as follows. First, based on the calculation of pooled relative uncertainty introduced in Eqs. (3) and (4) in the previous study^14^, the following equation gives the uncertainty of a calibration curve *u*(*e*_*curve*_):

17 $$\begin{array}{c}u({e}_{curve})=\overline{{n }_{Q}}\sqrt{\frac{\sum_{i=1}^{k}({N}_{rw,i}-1)\left[{\left(\frac{u\left(\overline{{n }_{r,i}}\right)}{{R}_{\overline{n\_r,i}}}\right)}^{2}+{\left(\frac{{s}_{n\_rQ,i}/\sqrt{{N}_{rw,i}}}{\overline{{n }_{rQ,i}}}\right)}^{2}\right]}{\sum_{i=1}^{k}({N}_{rw,i}-1)}}\end{array}$$

In this equation, *e*_*curve*_ denotes an estimation of the error of the mean quantification result sourced from the calibration curve, $\overline{{n }_{Q}}$ is the mean PCR quantification result of target sample replicates; *N*_*rw,i*_ is the effective number of wells containing calibrators with the same *i*-th representative DNA copy number; ${R}_{\overline{n\_r,i}}$ is the representative value of an estimate of the mean DNA copy number of these calibrators $\overline{{n }_{r,i}}$ defined in previous sub-sections; $u\left(\overline{{n }_{r,i}}\right)$ is the symmetric uncertainty of $\overline{{n }_{r,i}}$ calculated in previous sub-sections; and *s*_*n_rQ,i*_ and $\overline{{n }_{rQ,i}}$ are the corrected sample standard deviation and mean PCR quantification results for calibrator replicates. Finally, based on equations (S1) and (S3) in Supplementary Information File 2, the symmetric expanded uncertainty of the mean DNA copy number of target samples in each well, *U*_*conv*_, was calculated with the following equation:

18 $$\begin{array}{c}{U}_{conv}=\sqrt{{\left({t}_{0.05,{N}_{w}-1}\frac{{s}_{n\_Q}}{\sqrt{{N}_{w}}}\right)}^{2}+{\left[2u\left({e}_{curve}\right)\right]}^{2}}\end{array}$$

In this equation, *s*_*n_Q*_ is the corrected sample standard deviation of real-time PCR quantification results of target sample replicates, *N*_*w*_ is the number of wells containing target sample replicates including those that failed to amplify (undetermined), and ${t}_{0.05,{N}_{w}-1}$ is the two-tailed critical *t*-value introduced in Supplementary Information File 2. We considered the uncertainty of the calibration curve was irrelevant to the sample size. Therefore, the corresponding coverage factor was fixed at 2. The representative value of the mean quantification result of target samples in this method was $\overline{{n }_{Q}}$.

Wells with no amplification were excluded from the calculation of $\overline{{n }_{rQ,i}}$ and *s*_*n_rQ,i*_ because a calibration curve is a fitted line between calibrator Cq values and copy numbers. Failure to amplify indicates a copy number of 0 and should be included in the quantification results. Therefore, the quantification results for all wells were used to calculate $\overline{{n }_{Q}}$ and *s*_*n_Q*_. We compared the result with undetermined replicates defined as 0 to the results with these wells excluded.

Next, we evaluated asymmetric expanded uncertainties of the mean quantification result of target samples by calculating asymmetric confidence intervals. First, we calculated asymmetric expanded uncertainties of a mean PCR quantification result. DNA copy numbers converted from Cq values were assumed to follow the log-normal distribution^23–25^. In general, the confidence interval of a variable *X* that follows the log-normal distribution is calculated using the mean and corrected sample standard deviation of *Y* = ln(*X*)^26^. However, no Cq values exist for wells in which the copy number was 0. This indicates that PCR quantification results are not strictly log-normal distributed when zero-copy samples are included, and the mean and corrected sample standard deviation of *Y* are unavailable. However, most of the results with positive values are log-normal distributed. Thus, we concluded that the approximation based on the log-normal distribution was still a practical and useful method compared to other distributions. Therefore, the mean and corrected sample standard deviation of *Y* when zero-copy samples are included, $\overline{Y}$ and *s*_*Y*_, were estimated according to the relationship shown in Eqs. (11) and (12) in a previous study^27^, using the following equations:

19 $$\begin{array}{c}{s}_{Y}=\sqrt{\mathrm{ln}\left(\frac{{s}_{X}^{2}}{{\overline{X}}^{2}}+1\right)}\end{array}$$

20 $$\begin{array}{c}\overline{Y}=\mathrm{ln}\left(\overline{X}\right)-\frac{{s}_{Y}^{2}}{2}\end{array}$$

In these equations, $\overline{X}$ and *s*_*X*_ are the mean and corrected sample standard deviation of *X*, respectively. Based on confidence limits calculated with the modified Cox method^26^, ${A}_{\overline{X} }$ and ${B}_{\overline{X} }$, left- and right-side confidence intervals for $\overline{X}$ were calculated with the following equations:

21 $$\begin{array}{c}{A}_{\overline{X} }={M}_{X}-\mathrm{exp}\left(\overline{Y}+\frac{{s}_{Y}^{2}}{2}-{t}_{0.05,{N}_{X}-1}\sqrt{\frac{{s}_{Y}^{2}}{{N}_{X}}+\frac{{s}_{Y}^{4}}{2\left({N}_{X}-1\right)}}\right)\end{array}$$

22 $$\begin{array}{c}{B}_{\overline{X} }=\mathrm{exp}\left(\overline{Y}+\frac{{s}_{Y}^{2}}{2}+{t}_{0.05,{N}_{X}-1}\sqrt{\frac{{s}_{Y}^{2}}{{N}_{X}}+\frac{{s}_{Y}^{4}}{2\left({N}_{X}-1\right)}}\right)-{M}_{X}\end{array}$$

In these equations, *N*_*X*_ is the sample size of *X*; and *M*_*X*_ is the median of *X*, which is given by the following equation (see also equation 1.4 in reference^28^):

23 $$\begin{array}{c}{M}_{X}=\mathrm{exp}\left(\overline{Y}\right)\end{array}$$

In this study, the median was used as a representative real-time PCR quantification result because the median is closer to the peak of an asymmetric distribution than the mean. Note that *X* here corresponds to the PCR quantification result of DNA copy number of either a calibrator or a target sample.

Subsequently, left- and right-side confidence intervals for the expectation of the error of the mean result sourced from a calibration curve, *A*_*e_curve*_ and *B*_*e_curve*_, were calculated separately based on Eq. (17):

24 $$\begin{array}{c}{A}_{e\_curve}={M}_{Q}\sqrt{\frac{\sum_{i=1}^{k}\left({N}_{rw,i}-1\right)\left[{\left(\frac{{A}_{\overline{n\_r,i}}}{{R}_{n\_r,i}}\right)}^{2}+{\left(\frac{{A}_{\overline{n\_rQ,i}}}{{M}_{rQi}}\right)}^{2}\right]}{\sum_{i=1}^{n}\left({N}_{rw,i}-1\right)}}\end{array}$$

25 $$\begin{array}{c}{B}_{e\_curve}={M}_{Q}\sqrt{\frac{\sum_{i=1}^{k}\left({N}_{rw,i}-1\right)\left[{\left(\frac{{B}_{\overline{n\_r,i}}}{{R}_{n\_r,i}}\right)}^{2}+{\left(\frac{{B}_{\overline{n\_rQ,i}}}{{M}_{rQ,i}}\right)}^{2}\right]}{\sum_{i=1}^{n}\left({N}_{rw,i}-1\right)}}\end{array}$$

In these equations, *M*_*Q*_ is the estimated median of PCR quantification results of replicates of target samples calculated with Eq. (23); ${A}_{\overline{n\_r,i}}$ and ${B}_{\overline{n\_r,i}}$ are asymmetric expanded uncertainties of $\overline{{n }_{r,i}}$ calculated in previous sub-sections; *M*_*rQ,i*_ is the estimated median of PCR quantification results of replicates of corresponding calibrators calculated with Eq. (23); and ${A}_{\overline{n\_rQ,i}}$ and ${B}_{\overline{n\_rQ,i}}$ are the left- and right-side confidence intervals for the mean of the PCR replicates of the calibrators from Eqs. (21) and (22).

Finally, asymmetric expanded uncertainty of the mean DNA copy number of target samples, represented by left and right confidence intervals *A*_*prop*_ and *B*_*prop*_, is given by the following equations based on equation (S1):

26 $$\begin{array}{c}{A}_{prop}=\sqrt{{A}_{Q}^{2}+{A}_{e\_curve}^{2}}\end{array}$$

27 $$\begin{array}{c}{B}_{prop}=\sqrt{{B}_{Q}^{2}+{B}_{e\_curve}^{2}}\end{array}$$

In these equations, *A*_*Q*_ and *B*_*Q*_, are left- and right-side confidence intervals of the mean PCR quantification result of target sample replicates based on Eqs. (21) and (22). The representative value of the mean DNA copy number of target samples here was the median *M*_*Q*_.

We thus obtained symmetric and asymmetric expanded uncertainties of the mean DNA copy number of target samples in each well quantified by real-time PCR, *U*_*conv*_, and *A*_*prop*_ and *B*_*prop*_. The corresponding 95% confidence intervals were [$\overline{{n }_{Q}}$ − *U*_*conv*_, $\overline{{n }_{Q}}$* + U*_*conv*_] and [*M*_*Q*_ – *A*_*prop*_, *M*_*Q*_ + *B*_*prop*_].

### Calculation results for the uncertainty of reference materials

The upper half of Table 2 shows the uncertainty calculation results using the conventional method and interval widths on both sides of the representative value of 95% confidence interval calculated by the proposed method for the calibrator prepared by the serial dilution method (the number of wells in which the calibrator at each concentration amplified is shown in Table 3). Note that the symmetric expanded uncertainties of mean DNA copy number of calibrators shown in Table 2, *U*_*r*_, are simply two times of the corresponding symmetric uncertainties. Table S1-1 in Supplementary Information File 1 shows parameters and results, such as uncertainty of the pipette, the uncertainty of concentration of the dilution series and interval widths on both sides of the representative value of 95% confidence interval, as well as interval widths on both sides of the representative value of confidence interval arising from deviation because of the Poisson distribution.

**Table 2:** Results of measurement uncertainty of calibrators.

Representative DNA copy number *R*_*r*_	Symmetric uncertainty of mean DNA copy number of replicates ${u}_{r}\left(\overline{{n }_{r}}\right)$	Symmetric expanded uncertainty of mean DNA copy number of replicates *U*_*r*_	Left-side confidence interval for mean DNA copy number of replicates *A*_*r*_	Right-side confidence interval for mean DNA copy number of replicates *B*_*r*_
**Dilution series**				
80	5.14	10.3	10.0	10.3
40	3.22	6.43	6.21	6.21
20	2.11	4.22	3.88	4.23
10	1.43	2.86	2.73	2.74
5	1.00	2.00	1.81	1.82
1	0.726	1.45	0.769	1.45
**New reference material**				
79	1.04	2.07	1.63	2.04
39	0.730	1.46	1.22	1.22
20	0.527	1.05	0.817	1.22
9	0.369	0.739	0.408	0.816
5	0.282	0.565	0.408	0.816
1	0.186	0.373	0.000	0.500

**Table 3:** Real-time PCR results of calibrators.

Representative DNA copy number *R*_*r*_	Mean Cq value of replicates $\stackrel{\mathrm{-}}{\text{rCq}}$	Mean DNA copy number of replicates quantified by the calibration curve $\overline{{n }_{rQ}}$	Corrected standard deviation of DNA copy number of replicates quantifed by the calibration curve *s*_*rQ*_	Estimated median of DNA copy number of replicates quantified by the calibration curve *M*_*rQ*_	Effective numbers of well excluded undetermined wells *N*_*rw*_
**Dilution series**					
80	31.5	88.2	14.6	87.0	6
40	32.5	44.6	4.57	44.4	6
20	33.7	20.4	4.95	19.8	6
10	35.2	7.75	3.04	7.22	6
5	35.8	5.84	3.33	5.07	6
1	37.5	1.69	0.998	1.45	2
**New reference material**					
79	31.8	80.5	3.01	80.4	6
39	32.8	38.5	2.66	38.4	6
20	33.8	19.5	0.585	19.5	6
9	34.9	9.345	0.883	9.30	6
5	35.8	5.16	1.60	4.93	6
1	38.0	1.04	0.291	1.00	4

Measurement results for factors that affect the uncertainty of the new reference material were as follows. The flow cytometry measurement results of the intracellular DNA copy number revealed that the proportion of cell aggregates with a DNA copy number of 2 was 1.29%. The aggregation rate of the cell suspension was 1.17%. Therefore, the measured proportion of single cells with a DNA copy number of 2 was 0.12%, and the worst value was 0.144%. Regarding the aggregation rate of the cell suspension for inkjet dispensing, the number of aggregated masses was 45 of 2553 cell masses; therefore, the worst aggregation rate was 2.37%. The measurement results of APD detection and false detection rates revealed that 1043 of 1047 droplets contained one cell aggregate. Moreover, seven droplets had no cell aggregates; therefore, the worst detection and false detection rates were 99.0% and 1.43%, respectively. The APD measurement results for inkjet ejection of the cell suspension showed that particles were detected in 876 of 14,479 droplets, and thus the worst particle concentration in droplets was 0.0639. Further, in contamination evaluation by real-time PCR, all negative controls were undetermined; moreover, among 80 remaining wells, amplification occurred in one well, but the Cq value was 45.0. Because the mean Cq value + 6*s* for the sample with a copy number of 1 was ≤ 40 in this experiment; therefore, we concluded that specific amplification did not occur, and the number of DNA contaminants was 0. In other words, the worst probability of DNA contamination from reagents and the environment was 0.0571%. Finally, the distribution of the DNA copy number of the new reference material was combined from these components. The lower half of Table 2 shows the uncertainty calculation results using the conventional method and interval widths on both sides of the representative value of a 95% confidence interval calculated using the proposed method. Overall, the uncertainty was lower than that for the calibrator prepared by serial dilution. Moreover, strong asymmetry was reported when the copy number was 1, 5, 9, 20, and 79. When the copy number was 39, the asymmetry was rounded during the calculation process of the minimum integer interval of production variability.

### Real-time PCR quantification results and measurement uncertainty estimation results

**Figure 1:**
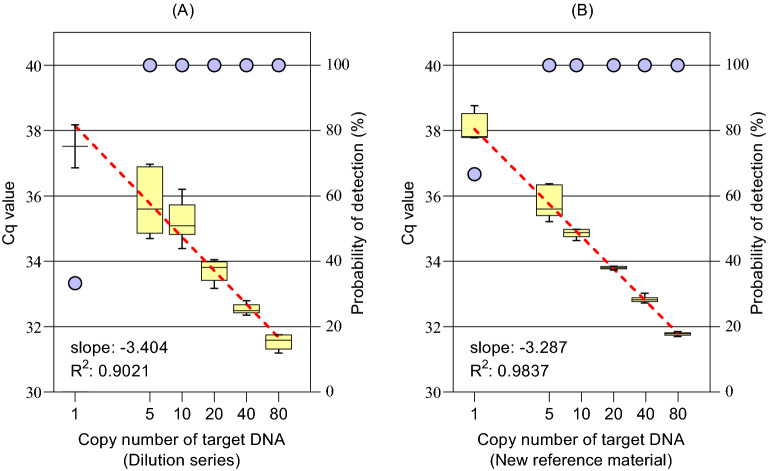
Box plots of real-time PCR results and standard curves (red dashed lines) for (**A**) calibrator prepared by the serial dilution method and (**B**) calibrator prepared using the new reference material. A whisker in the box plots shows the 10th and 90th percentiles. A purple circle indicates the percentage of wells in which the calibrator of each copy number amplified (probability of detection).

Table 3 and Fig. 1 show real-time PCR results for two different calibrators. All negative controls did not show amplification. The slope, intercept, and coefficient of determination (R^2^) of the calibration curve prepared by the serial dilution method were − 3.40, 38.1, and 0.902, respectively; therefore, the amplification efficiency was 96.7%. Moreover, the slope, intercept, and R^2^ of the calibration curve prepared with the new reference material were − 3.29, 38.0, and 0.984, respectively. Therefore, the amplification efficiency was 101%. The relative uncertainty $u({e}_{curve})/\overline{{n }_{Q}}$ of the calibration curve prepared with dilution series, *A*_*e_curve*_/*M*_*Q*_, and *B*_*e_curve*_/*M*_*Q*_ were 0.247, 0.414, and 62.3, respectively. The relative uncertainty $u({e}_{curve})/\overline{{n }_{Q}}$ of the calibration curve prepared with the new reference material, *A*_*e_curve*_/*M*_*Q*_, and *B*_*e_curve*_/*M*_*Q*_ were 0.101, 0.166, and 0.340, respectively. For the calibration curve prepared with the dilution series, the one-copy sample amplified in only two wells, and thus the two-tailed t critical value increased to 12.7. Therefore, the relative uncertainty on the right side became very large.

Next, Tables 4 and 5 shows quantification results for the target sample with two different calibration curves. Among the six wells, the result was undetermined for two wells and the probability of detection (POD) of the target sample was 66.7%. Cq values for the wells in which amplification occurred were 37.8, 36.66, 37.8, and 36.8. The copy number quantification results with the calibration curve prepared with the dilution series were 1.24, 2.74, 1.28, and 2.44, whereas the copy number quantification results with the calibration curve prepared with the new reference material were 1.17, 2.64, 1.20, and 2.35, respectively. Quantification results for undetermined wells were treated as zero in Table 4.

**Table 4:** Real-time PCR results of target samples to be quantified including undetermined wells as 0 copy.

Mean DNA copy number of replicates $\overline{{n }_{Q}}$	Corrected sample deviation of DNA copy number of replicates *s*_*Q*_	Estimated median of DNA copy number of replicates *M*_*Q*_	Left-side confidence interval for mean DNA copy number of replicates *A*_*Q*_	Right-side confidence interval for mean DNA copy number of replicates *B*_*Q*_
**Calibrated by the dilution series**				
1.28	1.16	0.951	0.453	2.36
**Calibrated by the new reference material**				
1.23	1.12	0.906	0.434	2.28

**Table 5:** Real-time PCR results of target samples to be quantified excluding undetermined wells.

Mean DNA copy number of replicates $\overline{{n }_{Q}}$	Corrected sample deviation of DNA copy number of replicates *s*_*Q*_	Estimated median of DNA copy number of replicates *M*_*Q*_	Left-side confidence interval for mean DNA copy number of replicates *A*_*Q*_	Right-side confidence interval for mean DNA copy number of replicates *B*_*Q*_
**Calibrated by the dilution series**				
1.93	0.777	1.79	0.779	1.90
**Calibrated by the new reference material**				
1.84	0.768	1.70	0.809	1.99

The final measurement uncertainty was calculated with Eqs. (17)–(27). When the calibration curve prepared with the dilution series was used for quantification, the quantification result with the expanded uncertainty for the target sample calculated by the conventional method was 1.28 ± 1.38. Therefore, the 95% confidence interval was [– 0.092, 2.66]. Moreover, the quantification result calculated by the proposed method was 0.951, and the 95% confidence interval was [0.350, 60.3]. When the calibration curve prepared with the new reference material was used for quantification, the quantification result with the expanded uncertainty for the target sample calculated by the conventional method was 1.23 ± 1.20. Therefore, the 95% confidence interval was [0.025, 2.43]. Moreover, the quantification result calculated by the proposed method was 0.906, and the 95% confidence interval was [0.447, 3.21].

For comparison, we also calculated the results after excluding wells in which the target failed to amplify. When the calibration curve prepared with the dilution series was used for quantification, the result with the expanded uncertainty for the target sample calculated by the conventional method was 1.93 ± 1.56. Therefore, the 95% confidence interval was [0.364, 3.49]. The quantification result calculated by the proposed method was 1.79, and the 95% confidence interval was [0.711, 113]. When the calibration curve prepared with new reference material was used, the result with the expanded uncertainty for the target sample calculated by the conventional method was 1.84 ± 1.28. Therefore, the 95% confidence interval was [0.564, 3.12]. The quantification result calculated by the proposed method was 1.70, and the 95% confidence interval was [0.891, 3.69].

## Discussions

In this paper, we describe models for calculating asymmetric uncertainties in measurements obtained by real-time PCR quantification. We separately evaluated left- and right-side uncertainties of two kinds of serially diluted and new reference material produced by an inkjet system and real-time PCR. We considered the influence of the Poisson distribution as a significant component in the dilution series model and proposed a new model for calculating the production variability of new reference material. We also introduced several factors for consideration in modeling real-time PCR quantification. The dilution series models were based on the general models given in Guide to the Expression of Uncertainty in Measurement^18^. We also used the concept to combine the relative uncertainties of several components described previously^14^.

The uncertainty evaluation results for the one-copy calibrator by the proposed method showed similar tendencies with the dilution series and the new reference material. When the dilution series were used, the sample’s confidence interval with a copy number of 1 was strongly asymmetric with respect to the representative value because the Poisson distribution with *λ* = 1 is strongly asymmetric. When the new reference material was used, the distribution was strongly asymmetric for the one-copy sample because it follows a peculiar distribution pattern. When the copy number was ≥ 5, the calibrator’s uncertainty prepared by serial dilution was generally symmetric because the Poisson distribution’s asymmetry was very weak. However, a certain level of asymmetry was seen for the calibrator prepared with the new reference material even when the copy number was ≥ 5 because of the characteristics of the distribution. Such a measurement result with asymmetric uncertainty can be separately expressed with the representative value *N* and the confidence interval [*L*, *H*] as “*N*, [*L*, *H*]”. Moreover, it can be expressed with the representative value and distances from the representative value to the lower and upper limits of the confidence interval, *A* and *B*, respectively, like ${N}_{-A}^{+B}$.

The mean copy number’s relative uncertainty was considerable for the dilution series because the Poisson distribution has a dominant effect when the copy number is ≤ 80. Similarly, the Poisson distribution has certain impact on the new reference material depending on the concentration of the cell suspension used. Nevertheless, the effect was minor because the cell suspension concentration was very low. Overall, the new reference material had low uncertainty.

Furthermore, the POD results for the one-copy calibrators showed an enormous difference. While the POD for the new reference material was 66.7%, the POD for the dilution series was 33.3%. These results suggest that the calibrator prepared with the dilution series does not contain DNA at a high probability. Note that the one-copy calibrator was prepared with the same diluted solution used to prepare target samples. However, the sample size was small and the value of POD varied widely. Moreover, the variability of Cq values was relatively high for the dilution series. This arises from the high uncertainty of the calibrator itself and the operator's work variability; however, the new reference material is associated with relatively low uncertainty and is less susceptible to the operator effect. Therefore, relatively highly precise calibration curves can be obtained with the new reference material.

The amplification efficiency for the new reference material’s calibration curve exceeded 100%, presumably because the confidence interval for the low-copy-number calibrator was skewed toward the right. The copy number of a low-copy-number calibrator is possibly larger than the representative value. Moreover, the calibration curve was an approximated plot of data with representative values. Therefore, the left side of the calibration curve was lowered, and the slope’s absolute value became smaller. The lower limit of the confidence interval estimated by the conventional method was a negative value for quantifying the target sample with both reference materials. However, the estimation results with the proposed method were always ≥ 0. As the DNA copy number cannot be a negative value, in reality, the negative part of the confidence interval has no physical meaning. Therefore, the proposed method can allow for more realistic uncertainty estimation. Moreover, for the dilution series, the asymmetry of the Poisson distribution has relatively strong effects when the copy number is low. Therefore, the actual copy number is possibly larger than the calculated value. When the new reference material is used, the actual copy number is likely to be larger than the calculated value because of the effects of cell aggregation and false-negative results of APD detection. Therefore, the calibration curve is likely to shift to the right from the approximation result. In other words, even when the Cq value was the same, it is likely to be larger than the calculated copy number of the calibrated sample to be quantified. This is another reason why uncertainty estimation with the proposed method is more appropriate.

Moreover, when the dilution series was used for quantification, the relative uncertainty on the right side was substantial because the POD of the one-copy calibrator was low. Therefore, the uncertainty on the right side estimated by the new method was very large, suggesting that copy number quantification with low POD is inappropriate.

Subsequently, when excluding wells in which the target failed to amplify, the mean DNA copy number increased to nearly 2, although the expectation was 1. One possible reason for this discrepancy is that the concentration of the diluted solution drifted, creating a large uncertainty in the one-copy solution. However, it is also possible that a one-copy sample, once diluted, would contain no DNA. Therefore, it is appropriate to include the zero-copy wells.

Furthermore, the uncertainty of the calibrator used is added to the measurement uncertainty. The use of reference material with smaller uncertainty improved the measurement precision of real-time PCR. Assay optimization, such as internal control and an increased number of samples, is necessary to reduce the uncertainty further. Moreover, the quantifiable range of copy numbers may be expandable using a calibrator that is excellent in terms of uncertainty in low copy numbers, such as 1 copy and 5 copies, and POD.

Currently, the coefficient of variation (CV) of quantification results is used to evaluate the LOQ of real-time PCR^29,30^. However, the calibrator uncertainty and bias of the uncertainty of real-time PCR in a low-copy-number region have effects on measurement results. Therefore, these factors should be considered. Moreover, criteria for evaluation of LOD include the percentage of positive replicates (equivalent to POD)^29,30^; however, considerations are required for copy number results of zero, which are included in measurement results, and the variability of the sample itself. Therefore, how to reflect these effects in the definition of LOD is considered to be an important issue.

## Methods

We prepared low-copy-number calibrators by the serial dilution of certified reference material (6205-a DNA600-G, National Metrology Institute of Japan) with an initial DNA copy number concentration of 2.24 × 10^9^ μL^−1^. The dilution protocol is shown in Table 6. The dilution buffer was prepared by mixing 1480 μL of 1× TE buffer (TE, pH 7.0, RNase-free, Thermo Fisher Scientific), 1480 μL of UltraPure DNase/RNase-Free Distilled Water (Thermo Fisher Scientific), and 40 μL of ColE1 DNA (318-00436, 450 ng μL^−1^, NIPPON GENE). ColE1 DNA was added to prevent DNA containing the target sequence from adhering to the inner wall of wells.

**Table 6:** Protocol of serial dilutions.

Concentration after diluted *C*_*after*_ (μL^−1^)	Volume of previous dilution *p* (μL)	Volume of buffer *q* (μL)
2.24 × 10^9^		
2.00 × 10^7^	4.00	444
2.00 × 10^5^	4.00	396
2.00 × 10^3^	4.00	396
20.0	4.00	396
10.0	200	200
5.00	200	200
2.50	200	200
1.25	150	150
0.250	40.0	160

The forward and reverse primers and probes used for real-time PCR experiments were 5′-TCGAAGGGTGATTGGATCGG-3′, 5′-TGGCTAGCTAAGTGCCATCC-3′, and 5′-6-FAM-TGCATTCTGGCTTCGATTGTCCCTAC-TAMRA-3′, respectively. The size of the amplicon produced by the primers was 100 bp. A 100 μM primer solution (100 μL) was diluted to 10 μM primer by mixing with 1× TE buffer (900 μL). A 100 μM probe solution (20 μL) was diluted to 2 μM probe with 1× TE buffer (980 μL). The amplification reagent was prepared by mixing 1050 μL of GenCheck qPCR Probe Master (dUTP, FASMAC), 105 μL of 10 μM forward primer, 105 μL of 10 μM reverse primer, 210 μL of 2 μM probe, and 210 μL of UltraPure DNase/RNase-Free Distilled Water. Finally, 16 μL of the amplification reagent and 4 μL of a template DNA solution were added to each well. To construct a calibration curve, we dispensed two different calibrators, the dilution series, and the new reference material, into a 96-well plate. The solutions of each calibrator at six different concentrations (1, 5, 10, 20, 40, and 80 copies) were added to five wells each in rows B to F. As a negative control, the amplification reagent alone was added to 6 wells in row A of the plate. In addition, calibrators with copy numbers of 1, prepared by the serial dilution method, were added to six wells in row H as quantification targets.

QuantStudio 12K Flex (Applied Biosystems) was used to carry out real-time PCR measurements. The thermal cycling condition was: reaction at 50 °C for 2 min and at 95 °C for 10 min, followed by 50 cycles of reaction at 95 °C for 30 s and at 61 °C for 1 min. The threshold was fixed to 0.2. For target sample wells, the DNA copy number was set to zero when the real-time PCR result was undetermined.

The preparations of cell suspensions for preparation of the new reference material and treatments after dispensing, such as cell wall lysis, were performed as described in the previous study^13^, except that *Synthetic construct DNA 6203-a-G* (600-G, GenBank registration number AB610938.1), instead of *hmg-Le1*, was incorporated into yeast.

## Conclusions

This risk of false-negative results in real-time PCR detection of environmental or viral DNA is great, and the demand for improved measurement precision is significant. However, the spread and development of genetic testing remain unsatisfactory because of inadequate concentration precision of low-copy-number calibrators and the unavailability of methods for appropriate evaluation of the uncertainty of test results. With the advent of reference material with high precision in a low-copy-number region, genetic testing has come one step closer to a new stage. Furthermore, in this study, we showed a method to reflect the asymmetric character of the uncertainty of low-copy-number calibrators in the calculation and evaluate the uncertainty of real-time PCR measurements more appropriately. Combining these, real-time PCR quantification of samples from one copy, which should be theoretically possible, will become possible in the near future. Moreover, the conclusion that the confidence intervals of real-time PCR measurement results are skewed to the right is applicable to qualitative tests. It may contribute to improving the reliability of qualitative results in various applications such as tests to rule out viruses or mycoplasma.

## Supplementary Information

Supplementary Information 1.

Supplementary Information 2.

Supplementary Information 3.

## Data Availability

All data generated and/or analyzed during current study are available within the manuscript and the supplementary information file.
