# Supplementary material for: Evaluation method for asymmetric uncertainty of quantitative polymerase chain reaction measurements of deoxyribonucleic acids with low copy number
Source: Sci Rep. 2021 Jun 2;11:11550. doi: 10.1038/s41598-021-90959-0 (PMC8172552; doi:10.1038/s41598-021-90959-0)
Supplement: Supplementary file 1 — Supplementary Information 1. [file 41598_2021_90959_MOESM1_ESM.pdf]

Supplementary Information File 1

Evaluation Method for Asymmetric Uncertainty of Quantitative Polymerase Chain Reaction Measurements of Deoxyribonucleic Acids with Low Copy Number

Unoh Ki*, Takeru Suzuki, Satoshi Nakazawa, Yuuki Yonekawa, Kazuki Watanabe, Michie Hashimoto, Ikuo Katoh, Shigeo Hatada, Hirotaka Unno

Corresponding author.

* E-mail: unoh.ki@jp.ricoh.com

Table of contents

Figure S1. Schematic diagram of the improved inkjet and cell counting system

Figure S2. Flowchart of method for estimating the interval which contains variable *x* with a probability over 95%

Table S1-1. Parameters and Detailed Results of Measurement Uncertainty of Dilution Series

Table S1-2. Two-tailed critical value $t_{0.05,\nu}$ of t-distribution with degrees of freedom ν and confidence level 95%

Table S1-3. Cq values of calibrators and samples for quantification

Reference

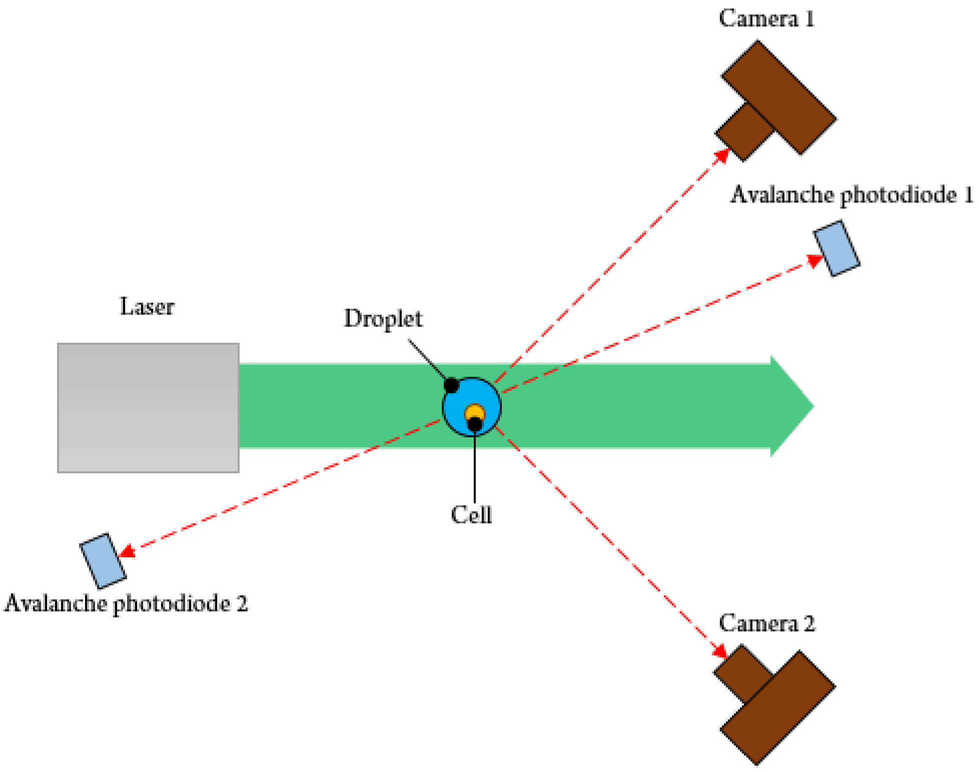

Figure S1 A schematic diagram of a counting system for the production of an improved reference material based on the system developed by Seo M. et al. 2019[1]. Two opposing avalanche photodiodes (APDs) were installed to detect the presence of cells in flying droplets. When the concentration of the cell suspension is very low, the probability of two or more cells existing in a droplet is low. Therefore, the number of droplets containing cells detected by APD was used as an estimated number of cells.

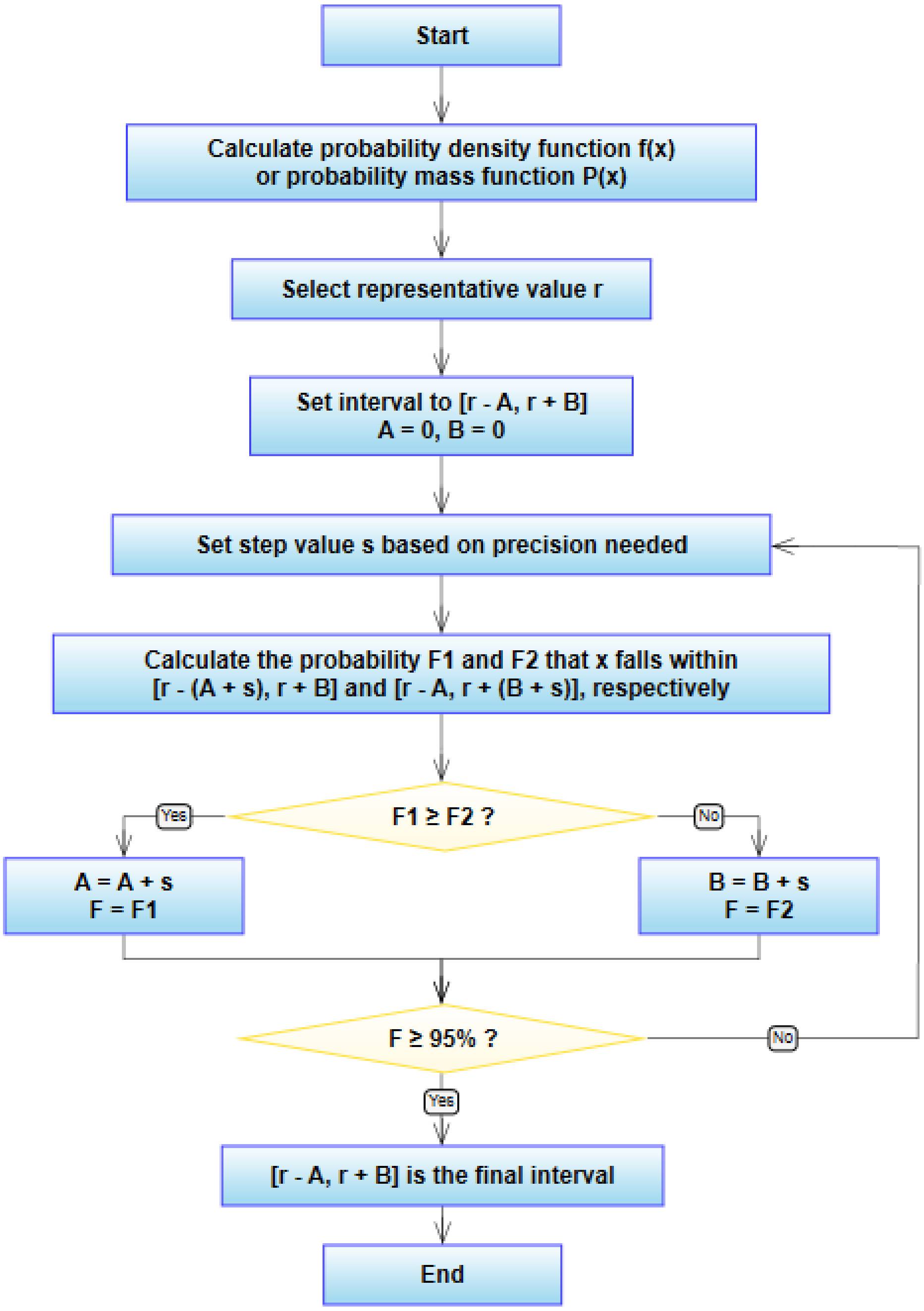

Figure S2 A flowchart of a method used for calculating an interval in which 95% of variable *x* fall. Representative value *r* may be chosen from among mean, median, mode, and other values as needed. Since variable *x* had to be an integer in the text, the representative value and step value *s* were set to an integer and 1, respectively.

Table S1-1 Parameters and Detailed Results of Measurement Uncertainty of Dilution Series

C_{after} (μL^{-1})	$u(p)$ (μL)	$u(q)$ (μL)	$u(p_{final})$ (μL)	$u(c_{after})$ (μL^{-1})	A_{c_after}	B_{c_after}	$u(e)$	A_e	B_e
2.24×10^9				9.75×10^7	1.95×10^8	1.95×10^8			
2×10^7	0.0095	0.25		8.72×10^5	1.74×10^6	1.74×10^6	9.47×10^4	1.86×10^5	1.86×10^5
2×10^5	0.0095	0.25		8.73×10^3	1.75×10^4	1.75×10^4	8.94×10^3	1.75×10^4	1.75×10^4
2×10^3	0.0095	0.25		87.5	175	175	894	1.75×10^3	1.75×10^3
20	0.0095	0.25	0.0095	0.904	1.81	1.81	89.4	175	175
10	0.055	0.055	0.0095	0.479	0.954	0.955	63.2	123	124
5	0.055	0.055	0.0095	0.264	0.524	0.526	44.7	87	88
2.5	0.055	0.055	0.0095	0.154	0.303	0.305	31.6	61	62
1.25	0.055	0.055	0.0095	0.100	0.195	0.198	19.4	37	38
0.25	0.03	0.055	0.0095	0.0407	0.0759	0.0805	7.07	13	14

Variables in Table S1-1 are defined as follows:

C_{after}: the DNA concentration of diluted solution after each dilution step

$u(p)$: the symmetric uncertainty of the expectation of the volume of the original solution at each dilution step

$u(q)$: the symmetric uncertainty of the expectation of the volume of the buffer added at each dilution step

$u(p_{final})$: the symmetric uncertainty of the expectation of the volume of the diluted solution dispensed into each well

$u(c_{after})$: the symmetric uncertainty of C_{after}

A_{c_after} and B_{c_after}: left and right side confidence intervals for the expectation of C_{after}

$u(e)$: Symmetric uncertainty of the error of DNA copy number in the P volume of the original solution due to the Poisson distribution at each dilution step

A_e and B_e: Left and right intervals for representing the asymmetric variety of the error of DNA copy number in the P volume of the original solution due to the Poisson distribution at each dilution step

Table S1-2 Two-tailed critical value $t_{0.05,v}$ of t-distribution with degrees of freedom v and confidence level 95%

v	1	2	3	4	5	6	7	8	9	10	11	12	13	14	15
$t_{0.05,v}$	12.7	4.30	3.18	2.78	2.57	2.45	2.36	2.31	2.26	2.23	2.20	2.18	2.16	2.14	2.13

v	16	17	18	19	20	21	22	23	24	25	26	27	28	29	30
$t_{0.05,v}$	2.12	2.11	2.10	2.09	2.09	2.08	2.07	2.07	2.06	2.06	2.06	2.05	2.05	2.05	2.04

Table S1-3 Cq values of calibrators and samples for quantification

Estimated copy number	Cq value					
	Well 1	Well 2	Well 3	Well 4	Well 5	Well 6
**Calibrators prepared with the dilutions series**						
80	31.71	31.47	31.19	31.75	31.76	31.34
40	32.44	32.35	32.51	32.48	32.64	32.79
20	33.49	33.17	34.05	33.97	33.67	33.95
10	35.58	34.39	34.95	34.97	35.21	36.20
5	35.48	36.97	36.88	34.69	35.71	34.91
1	38.17	Undetermined	36.85	Undetermined	Undetermined	Undetermined
**Calibrators prepared with the new reference material**						
79	31.85	31.81	31.79	31.78	31.75	31.70
39	32.72	32.78	32.84	32.81	33.02	32.83
20	33.74	33.80	33.81	33.77	33.85	33.84
9	34.63	34.90	34.98	34.98	34.79	34.87
5	35.21	36.37	36.34	35.46	35.56	35.64
1	Undetermined	Undetermined	37.83	38.76	37.80	37.77
**Target samples for quantification prepared with the dilutions series**						
1	37.82	36.66	Undetermined	37.78	Undetermined	36.82

Reference

1. Seo, M. *et al.* Novel Bioprinting Application for the Production of Reference Material Containing a Defined Copy Number of Target DNA. *Anal. Chem.* **91**, 12733–12740 (2019).
