# Supplementary material for: Evaluation method for asymmetric uncertainty of quantitative polymerase chain reaction measurements of deoxyribonucleic acids with low copy number
Source: Sci Rep. 2021 Jun 2;11:11550. doi: 10.1038/s41598-021-90959-0 (PMC8172552; doi:10.1038/s41598-021-90959-0)
Supplement: Supplementary file 2 — Supplementary Information 2. [file 41598_2021_90959_MOESM2_ESM.pdf]

Supplementary Information File 2

Evaluation Method for Asymmetric Uncertainty of Quantitative Polymerase Chain Reaction Measurements of Deoxyribonucleic Acids with Low Copy Number

Unoh Ki*, Takeru Suzuki, Satoshi Nakazawa, Yuuki Yonekawa, Kazuki Watanabe, Michie Hashimoto, Ikuo Katoh, Shigeo Hatada, Hirotaka Unno

Corresponding author.

* E-mail: unoh.ki@jp.ricoh.com

Table of contents

Measurement uncertainty and real-time PCR quantification 2
Definition of measurement uncertainty 2
Real-time PCR quantification 3
An alternative model of the symmetric uncertainty of serially diluted calibrators 4
Method to calculate the combined distribution of DNA copy number of the calibrator in each well prepared with a new reference material 6
References 9

Measurement uncertainty and real-time PCR quantification

Definition of measurement uncertainty

Measurement uncertainty (or just uncertainty) is a parameter of a measurement result that characterizes statistical dispersion[1]. Many components comprise statistical dispersion, such as the variability of results between duplicate measurements, generally characterized as a standard deviation. Deviations also occur within the instrument used to perform the measurement. Generally, a measurement instrument should be calibrated regularly and its uncertainty provided in a calibration certificate. The uncertainty of any reference materials should also be taken into account and can be obtained from the manufacturer's certificate or manual. Other components of uncertainty include environmental conditions and inter-operator variability.

The uncertainty of a measurement result is combined with the uncertainties of components that significantly influence the result. A general method for calculating the combined uncertainty is provided in Guide to the Expression of Uncertainty in Measurement (GUM)[1]. Assume that a measurand Y is not directly measured and is determined by N other uncorrelated quantities X_1, X_2,..., X_N. If all Xi are independent of each other, the combined uncertainty $u_C(y)$ of y, an estimate of the expectation of Y, is given by the following equation in GUM[1] (5.1.2, equation (10)):

$$u_C(y) = \sqrt{\sum_{i=1}^{N} \left(\frac{\partial f}{\partial x_i}\right)^2 u^2(x_i)} \quad \text{(S1)}$$

In this equation, x_i estimates the expectation of X_i; f expresses the relationship between Y and all X_i, and $u(x_i)$ is the uncertainty when X_i takes the value x_i. The coefficient $\partial f/\partial x_i$ is the value of $\partial f/\partial X_i$ when $X_i = x_i$. The coefficient, which is referred to as the sensitivity coefficient of X_i, indicates how significantly X_i variability of influences y.

However, if Xi are correlated, $u_C(y)$ is given by the following equation in GUM[1] (5.2.2, equation (16)):

$$u_C(y) = \sqrt{\sum_{i=1}^{N} \left(\frac{\partial f}{\partial x_i}\right)^2 u^2(x_i) + 2\sum_{i=1}^{N-1} \sum_{j=i+1}^{N} \frac{\partial f}{\partial x_i}\frac{\partial f}{\partial x_j} u(x_i)u(x_j)r(x_i, x_j)} \quad \text{(S2)}$$

In this equation, $r(x_i, x_j)$ is the correlation coefficient of X_i and X_j.

Furthermore, an expanded uncertainty U is defined to indicate that with a confidence level (usually 95%), the expectation of Y lies within a symmetric confidence interval given by $[y - U, y + U]$. In most cases, Y is assumed to follow a normal distribution. Therefore, U is given by the following equation in GUM[1] (G.3.2, equation (G.1d)):

$$U = ku_C(y) = t_{\alpha,\nu}u_C(y) \quad \text{(S3)}$$

In this equation, the parameter k is referred to as the coverage factor. The student's two-tailed critical value $t_{\alpha,\nu}$ is generally used as the coverage factor (see Table S2-2). The critical value is determined

by a significance level α (confidence level equals $100(1-\alpha)\%$) and degrees of freedom υ (equals $n-1$ in general cases, where n is the sample size). The term "two-tailed" indicates that both upper and lower limits should be considered. In some applications, k is fixed at 2 for convenience. However, because $t_{\alpha,\upsilon}$ significantly exceeds two when n is small (>2.5 when n is ≤6), U will be significantly smaller when k is fixed at 2 but not $t_{\alpha,\upsilon}$, which means the confidence level will not be met. Therefore, we recommend $t_{\alpha,\upsilon}$. Nevertheless, the uncertainty provided by a certificate of a measurement instrument or certified reference material is not a result evaluated by users. Thus we assumed that it is a property of the population but not samples, and $k = 2$ is appropriate when calculating the expanded uncertainty of a measurement instrument or certified reference material.

Real-time PCR quantification

Theoretically, real-time PCR amplifies DNA copy number exactly two-fold per cycle, assuming an amplification efficiency E of 100%. Fluorescence excitation occurs during amplification, and the intensity is directly proportional to the copy number. However, in practice, E is unknown but less than 100%. Therefore, calibrators of known DNA copy number are used to quantify an unknown sample indirectly.

Fluorescence intensity is measured and plotted against the cycle number as the calibrator or sample to be quantified is amplified. A continuous amplification curve is then calculated. The cycle number at which the fluorescence intensity equals a selected threshold is referred to as a Cq. Next, a continuous calibration curve is obtained by plotting calibrator Cq values against the logarithm of their copy number. Finally, the copy number of the sample to be quantified is determined by its Cq value and the calibration curve. The amplification occasionally fails, especially when quantifying an extremely low-copy-number target sample because the actual DNA copy number may be zero. In these cases, the Cq value is undetermined.

Although the copy number and actual cycle numbers are integers, Cq values are continuous parameters because the amplification curve is continuous. Moreover, an estimation of copy number converted from a Cq value is also continuous because of the continuous calibration curve. Generally, a Cq value is assumed to follow a normal distribution. Thus, the estimation of copy number converted from a Cq value is assumed to follow a log-normal distribution[2–4].

Generally, a calibrator is prepared by serial dilution. A specific volume of an original DNA solution is diluted with a specific volume of buffer. The calibrator DNA is serially diluted to a known target concentration. Finally, a specific volume of diluted solution with a specific low concentration is dispensed into each well.

Moreover, a new reference material is developed to dispense cell suspension into each well by a inkjet system[5]. The number of fluorescent stained cells in each droplet ejected is counted by several light sensors. The total DNA copy number is controlled by the number of cells.

An alternative model of the symmetric uncertainty of serially diluted calibrators

Here, we describe another way to model the symmetric uncertainty of the mean DNA copy number of serially diluted calibrators prepared by dispensing diluted solution of a specific concentration into each well. After preparing the dilution series, 4 μL of each concentration was dispensed into a 96-well plate with a pipette. The mean final copy number N_{dil} in each well $\overline{N_{dil}}$ can be described using the following equation, corresponding to f in equation (S1):

$$\overline{N_{dil}} = \frac{1}{N_{rw}} \sum_{i=1}^{N_{rw}} P_{final,i} \cdot C_{final,i} + E_{final,i} \tag{S4}$$

In this equation, N_{rw} is the effective number of wells containing calibrators prepared by dispensing a specific diluted solution; $C_{final,i}$ (unit: μL^{-1}) is the concentration of the solution to be dispensed into the i-th well; $P_{final,i}$ (unit: μL) is the volume of solution dispensed in the i-th well, which was identically measured as 4 μL; and $E_{final,i}$ is the error of DNA copy number in the i-th well. The probability mass function of $E_{final,i}$ was defined by $P_{E\_final,i}(E_{final,I} = e_{final,i}) = P_{\lambda\_final,i}(X_i = e_{final,I} + \lambda_{final,i})$, where $e_{final,i}$ is a certain value of $E_{final,i}$; and $P_{\lambda\_final,i}$ is the probability mass function of a Poisson distributed variable X_i with an expectation of $\lambda_{final,I} = p_{final,I} \cdot c_{final,i}$, respectively. Here, $p_{final,i}$ and $c_{final,i}$ are estimates of the expectations of $P_{final,i}$ and $C_{final,i}$, respectively.

Consider that the $p_{final,i}$ volume of the diluted solution was obtained from the same container. Because $p_{final,i}$ was much smaller than the remaining volume of the diluted solution in the container. $c_{final,i}$ was assumed to remain precisely the same while dispensing. Thus, the correlation coefficient $r(c_{final,i}, c_{final,j})$ of each $c_{final,i}$ and $c_{final,j}$ pair corresponding to different wells was exactly 1. Moreover, the same $p_{final,i}$ volume of diluted solution was measured and dispensed with the same pipette. Thus, the error between $p_{final,i}$ and the true value of $P_{final,i}$ was assumed to be identical. Therefore, the correlation coefficient $r(p_{final,i}, p_{final,j})$ of each pair of $p_{final,i}$ and $p_{final,j}$ corresponding to different wells was exactly 1. Other pairs of variables, such as $p_{final,i}$ and $c_{final,i}$, or $e_{final,i}$ and $e_{final,j}$ corresponding to different wells, were assumed to be independent of each other.

Therefore, based on equation (S2), the uncertainty of the mean of the final copy number in each well $u(\overline{n_{dil}})$ was the positive square root of the combined variance $u^2(\overline{n_{dil}})$ given by the following equation:

$$
\begin{aligned}
u^2(\overline{n_{dil}}) &= \sum_{i=1}^{N_{rw}}\left[\frac{\partial\overline{n_{dil}}}{\partial c_{final,i}}u(c_{final,i})\right]^2+\sum_{i=1}^{N_{rw}}\left[\frac{\partial\overline{n_{dil}}}{\partial p_{final,i}}u(p_{final,i})\right]^2+\sum_{i=1}^{N_{rw}}\left[\frac{\partial\overline{n_{dil}}}{\partial e_{final,i}}u(e_{final,i})\right]^2\\
&+2\sum_{i=1}^{N_{rw}-1}\sum_{j=i+1}^{N_{rw}}\frac{\partial\overline{n_{dil}}}{\partial c_{final,i}}\frac{\partial\overline{n_{dil}}}{\partial c_{final,j}}u(c_{final,i})u(c_{final,j})r\left(u(c_{final,i}),u(c_{final,j})\right)\\
&+2\sum_{i=1}^{N_{rw}-1}\sum_{j=i+1}^{N_{rw}}\frac{\partial\overline{n_{dil}}}{\partial p_{final,i}}\frac{\partial\overline{n_{dil}}}{\partial p_{final,j}}u(p_{final,i})u(p_{final,j})r\left(u(p_{final,i}),u(p_{final,j})\right)\\
&= N_{rw}\left[\frac{p_{final}}{N_{rw}}u(c_{final})\right]^2+N_{rw}\left[\frac{c_{final}}{N_{rw}}u(p_{final})\right]^2+N_{rw}\left[\frac{1}{N_{rw}}\sigma_{E\_final}\right]^2\\
&+N_{rw}(N_{rw}-1)\left[\frac{p_{final}}{N_{rw}}u(c_{final})\right]^2+N_{rw}(N_{rw}-1)\left[\frac{c_{final}}{N_{rw}}u(p_{final})\right]^2\\
&= \left[p_{final}u(c_{final})\right]^2+\left[c_{final}u(p_{final})\right]^2+\frac{1}{N_{rw}}\sigma_{E\_final}^2 \qquad \text{(S5)}
\end{aligned}
$$

In this equation, $\overline{n_{dil}}$ is the estimate of the expectation of $\overline{N_{dil}}$; c_{final} and p_{final} denote identical values of $p_{final,i}$ and $c_{final,i}$ in all wells, respectively; $u(c_{final,i})$, $u(p_{final,i})$, and $u(e_{final,i})$ are variable uncertainties. Note that items with a correlation coefficient of zero were omitted. The uncertainty $u(c_{final,i})$ in each well was equal to $u(c_{final})$, which corresponds to the uncertainty of the concentration of the diluted solution calculated using equation (2) in the manuscript. Moreover, $u(p_{final,i})$ in each well was equal to $u(p_{final})$, which corresponds to the uncertainty of the pipette. The value of $u(p_{final})$ can be obtained directly from the calibration certificate. Furthermore, N_{dil} in each well independently and identically followed the Poisson distribution with an expectation $\lambda_{final} = p_{final} \cdot c_{final}$. Therefore, $u(e_{final,i})$ was equal to the standard deviation of the Poisson distribution $\sigma_{E\_final} = \sqrt{p_{final} \cdot c_{final}}$.

Method to calculate the combined distribution of DNA copy number of the calibrator in each well prepared with a new reference material

The distribution of the DNA copy number of the calibrator in each well prepared with a new reference material, N_{DNA}, can be obtained by combing the distributions of influential factors of the production variability. The relationship between N_{DNA} and influential factors can be represented as follows. The mean value of N_{DNA}, $\overline{N_{DNA}}$, is given by:

$$\overline{N_{DNA}} = N_{APD} \cdot [(\overline{N_{pa}} \cdot \overline{N_{agg\_pa}}) \cdot (\overline{N_{sgl\_agg}} \cdot \overline{N_{intra}})] + \overline{N_{con}} \qquad \text{(S6)}$$

In this equation, the constant N_{APD} is the predetermined number of APD detections in each well set by an operator in the inkjet system, $\overline{N_{pa}}$ is the mean number of particles dispensed between two consecutive APD detections, $\overline{N_{agg\_pa}}$ is the mean number of cell aggregates in a fluorescent particle (a fluorescent particle is either a cell aggregates or a fluorescent contaminant), $\overline{N_{sgl\_agg}}$ is the mean number of cells in a cell aggregate, $\overline{N_{intra}}$ is the mean intracellular DNA copy number and $\overline{N_{con}}$ is the mean contaminant DNA copy number per well from the reagents and environment.

Table S2-1 Combine the distributions of influential factors of production variability

Step No.	Variable X_i at i-th step	Variable Y_i at i-th step	Obtained combined variable Z_i at i-th step
Step 1	N_{pa}: the number of particles dispensed between two consecutive APD detections ($N_{pa} \geq 1$)	N_{agg_pa}: the number of cell aggregates in a fluorescent particle (N_{agg_pa} = 0 or 1)	N_{agg_APD}: the number of cell aggregates dispensed between consecutive APD detections
Step 2	N_{sgl_agg}: the number of single cells in a cell aggregate (N_{sgl_agg} = 1 or 2)	N_{intra}: the intracellular DNA copy number (N_{intra} = 1 or 2)	N_{DNA_agg}: the DNA copy number of a cell aggregate
Step 3	N_{pa_agg} (Step 1): the number of cell aggregates dispensed between two consecutive APD detections ($N_{pa\_agg} \geq 0$)	N_{DNA_agg} (Step 2): the DNA copy number of a cell aggregate ($1 \leq N_{DNA\_agg} \leq 4$)	N_{DNA_APD}: the copy number of DNA dispensed between two consecutive APD detections
Step 4	N_{APD}: the predetermined number of APD detections in each well set in the inkjet system (N_{APD} is a constant ≥ 1)	N_{DNA_APD} (Step 3): the copy number of DNA dispensed between two consecutive APD detections ($N_{DNA\_APD} \geq 0$)	N_{DNA_inkjet}: the DNA copy number dispensed in a well by the inkjet system after APD detected the existence of particles in a droplet N_{APD} times
Step 5	N_{DNA_inkjet} (Step 3): the copy number of DNA dispensed in a well by the inkjet system after APD detected the existence of particles in a droplet N_{APD} times ($N_{DNA\_inkjet} \geq 0$)	N_{con}: the copy number of DNA contaminant in a well from reagents and environment (N_{con} = 0 or 1)	N_{new}: the DNA copy number of the calibrator prepared with the new reference material in each well

The combined distribution of N_{DNA} is calculated as shown in Table S2-1. Steps 1–4 correspond to each multiplication relationship in equation (S6). Therefore, the calculations of the combined

distributions at Steps 1–4 were performed as follows.

At each step, X_i at the i-th step denotes the number of a kind of entity (referred to as entity A) existing in a specific range, such as particles in several droplets or cells in an aggregate. Y_i at the i-th step denotes the number of a second kind of entity (referred to as entity B) contained in entity A, such as the number of cell aggregates in a particle or the DNA copy number in a cell. For each of the entities A, Y_i independently takes a particular value. Consider that $y_{i,1}$, $y_{i,2}$, …, $y_{i,k}$ denote all possible values of Y_i. Therefore, a condition limited by a certain value of X_i, x_i, is satisfied according to the following condition:

$$x_i = \sum_{j=1}^{k} n_{i,j} \tag{S7}$$

In this equation, $n_{i,j}$ is the value of the number of entities A, $N_{i,j}$, of which Y_i takes the same value $y_{i,j}$. Y_i of all entities A can take the same $y_{i,j}$. Therefore, $N_{i,j}$ ranges from 0 to x_i. Note that X_i has a limited number of possible values (Table S2-1). Therefore, the variety of patterns of $N_{i,1}$, $N_{i,2}$,..., $N_{i,k}$ is limited.

Next, consider a specific pattern that X_i, $N_{i,1}$, $N_{i,2}$,..., $N_{i,k}$ take certain values x_i, $n_{i,1}$, $n_{i,2}$,..., $n_{i,k}$, which satisfies equation (S7). The probability of the pattern occurring is given by the following equation:

$$\begin{aligned} P_{X_i,N_{i,1},N_{i,2},\ldots,N_{i,k}}(x_i, n_{i,1}, n_{i,2}, \ldots, n_{i,k}) &= \Pr(X_i = x_i, N_{i,1} = n_{i,1}, N_{i,2} = n_{i,2}, \ldots, N_{i,k} = n_{i,k}) \\ &= \prod_{j=1}^{k} \binom{x - \sum_{l=0}^{j-1} n_{i,l}}{n_{i,j}} \Pr(Y_{i,j} = y_{i,j})^{n_{i,j}} \end{aligned} \tag{S8}$$

In this equation, the probability $\Pr(Y_{i,j} = y_{i,j})$ was measured and calculated as described in the manuscript. Consider that all x_i entities are distinguished from each other. Thus, the item $\binom{x-\sum_{l=0}^{j-1} n_{i,l}}{n_{i,j}}$ is the number of combinations indicating that specific $n_{i,j}$ entities were selected from the remaining $x - \sum_{l=0}^{j-1} n_{i,l}$ entities. Note that Y_i of an entity cannot take two values simultaneously. Therefore, once $n_{i,j}$ entities are selected, the remaining number of entities will decrease by $n_{i,j}$. Moreover, the denotation $n_{i,0}$ has no physical meaning. However, $n_{i,j}$ was defined as 0 for convenience.

Subsequently, the target variable at each step is the total number of entities B in all entities A, denoted by Z_i. The value of Z_i, z_i, corresponding to the specific pattern x_i, $n_{i,1}$, $n_{i,2}$,..., $n_{i,k}$, is given by the following equation:

$$z_i = \sum_{i=1}^{k} y_{i,j} \cdot n_{i,j} \tag{S9}$$

Note that Z_i and the pattern of X_i, $N_{i,1}$, $N_{i,2}$,..., $N_{i,k}$ are not bijective. Z_i corresponding to different patterns can take the same value. Therefore, the probability $\Pr(Z_i = z_i)$ is the sum of the probabilities of the occurrence of all patterns calculated by equation (S8), given that equations (S7) and (S9) are satisfied.

Furthermore, the number of patterns of X_i, $N_{i,1}$, $N_{i,2}$,..., $N_{i,k}$ can be enormous depending on the maximum value of X_i and the number of possible values of Y_i. Therefore, in an actual calculation, some variables were allowed to be within a limited range, and values with minimal probability were excluded from the calculation. Specifically, the range of N_{pa} at Step 1 was limited to [0, 10] (the probability to take a value out of the range was smaller than 1×10^{-16}), and the range of DNA copy number dispensed was limited to [0, 6] (the probability to take a value out of the range was smaller than 1×10^{-8}).

The above calculations were repeated at Steps 1–4 with corresponding X_i and Y_i defined in Table S2-1. Note that X_4 at Step 4 was the constant N_{APD} and has only one possible value. The combined distribution of Z_4 at Step 4 was used as the distribution of X_5 at Step 5. Step 5 corresponds to the additive relationship in equation (S6). The calculation of Step 5 was performed as follows. The probability mass function of the DNA copy number N_{new} of the calibrator in each well, $P_{N\_new}(n_{new})$, is given by the following equation:

$$P_{N\_new}(n_{new}) = \Pr(N_{new} = n_{new})$$
$$= \Pr\left(N_{DNA\_inkjet} = n_{new} - 1\right) \cdot \Pr(N_{con} = 1) + \Pr\left(N_{DNA\_inkjet} = n_{new}\right) \cdot \Pr(N_{con} = 0) \quad \text{(S10)}$$

In this equation, n_{new} is a specific value of N_{new}, N_{DNA_inkjet} is the total copy number of DNA dispensed by the inkjet system into each well, and N_{con} is the copy number of DNA contaminant from reagents and environment. N_{DNA_inkjet} was assumed to be independent of N_{con}. Note that possible values of N_{con} are 0 and 1.

Finally, the expectation of N_{new}, μ_{N_new}, and the standard deviation of N_{DNA}, σ_{N_new}, were calculated by using the following equations:

$$\mu_{N\_new} = \sum_{i}^{k} n_{new,k} \cdot P_{N\_new}\left(n_{new,k}\right) \quad \text{(S11)}$$

$$\sigma_{N\_new} = \sqrt{\sum_{i}^{k} \left(n_{new,k} - \mu_{N\_new}\right)^2 P_{N\_new}\left(n_{new,k}\right)} \quad \text{(S12)}$$

In these equations, $n_{new,1}$, $n_{new,2}$,..., $n_{new,\mathrm{k}}$ are all possibles values of N_{new}. Note that the probability mass function $P_{N\_new}(n_{new})$ was theoretically calculated based on measurement and estimation of several influence factors. Therefore, μ_{N_new} and σ_{N_new} are not strictly the expectation and standard deviation of the population. However, because precise measurement of DNA copy number remains undeveloped, we assumed μ_{N_new} and σ_{N_new} as the expectation and standard deviation of the population for convenience.

The above calculations yield the probability mass function, expectation, and standard deviation of the combined distribution of the DNA copy number of the calibrator in each well prepared with the new reference material.

References

1. JCGM 100:2008 Evaluation of measurement data – Guide to the expression of uncertainty in measurement.
2. Thompson, M. GMO Proficiency testing: Interpreting z-scores derived from log-transformed data. *AMC Tech. Br.* (2004).
3. Johnson, N. L., Kotz, S. & Balakrishnan, N. Lognormal Distributions. in *Continuous univariate distributions Vol.1* (ed. 2nd) xvi+387 (John Wiley & Sons, 1994).
4. Bland, J. M. & Altman, D. G. Statistics notes: Transformations, means, and confidence intervals. *BMJ* **312**, 1079–1079 (1996).
5. Seo, M. *et al.* Novel Bioprinting Application for the Production of Reference Material Containing a Defined Copy Number of Target DNA. *Anal. Chem.* **91**, 12733–12740 (2019).
